# The BBSome restricts entry of tagged carbonic anhydrase 6 into the *cis*-flagellum of *Chlamydomonas reinhardtii*

**DOI:** 10.1371/journal.pone.0240887

**Published:** 2020-10-29

**Authors:** Kewei Yu, Peiwei Liu, Dipna Venkatachalam, Brian M. Hopkinson, Karl F. Lechtreck

**Affiliations:** 1 Department of Cellular Biology, University of Georgia, Athens, Georgia, United States of America; 2 Department of Marine Sciences, University of Georgia, Athens, Georgia, United States of America; East Carolina University Brody School of Medicine, UNITED STATES

## Abstract

The two flagella of *Chlamydomonas reinhardtii* are of the same size and structure but display functional differences, which are critical for flagellar steering movements. However, biochemical differences between the two flagella have not been identified. Here, we show that fluorescence protein-tagged carbonic anhydrase 6 (CAH6-mNG) preferentially localizes to the *trans*-flagellum, which is organized by the older of the two flagella-bearing basal bodies. The uneven distribution of CAH6-mNG is established early during flagellar assembly and restored after photobleaching, suggesting that it is based on preferred entry or retention of CAH6-mNG in the *trans*-flagellum. Since CAH6-mNG moves mostly by diffusion, a role of intraflagellar transport (IFT) in establishing its asymmetric distribution is unlikely. Interestingly, CAH6-mNG is present in both flagella of the non-phototactic *bardet-biedl syndrome 1* (*bbs1*) mutant revealing that the BBSome is involved in establishing CAH6-mNG flagellar asymmetry. Using dikaryon rescue experiments, we show that the *de novo* assembly of CAH6-mNG in flagella is considerably faster than the removal of ectopic CAH6-mNG from *bbs* flagella. Thus, different rates of flagellar entry of CAH6-mNG rather than its export from flagella is the likely basis for its asymmetric distribution. The data identify a novel role for the *C*. *reinhardtii* BBSome in preventing the entry of CAH6-mNG specifically into the *cis*-flagellum.

## Introduction

Cilia function, structure and composition varies considerably between different species and cell types. Multicellular organisms, for example, possess cells with motile cilia, primary cilia and specialized sensory cilia such as the outer segment of photoreceptor neurons [1]. During development, mammalian epithelial cells, such as those of the ependyma, first assemble a single nonmotile cilium (hence the term primary cilium), followed by the development of numerous motile cilia [2]. In many unicellular organisms, distinct types of motile cilia or flagella are present concomitantly on a given cell. In ciliates such a *Tetrahymena*, the motile cilia in the oral apparatus capture food particles whereas those covering the rest of the cell body serve in cell locomotion. Most heterokonts possess a long flagellum with tripartite mastigonemes to provide propulsion and a short smooth flagellum possibly functioning as a ruder [3, 4]. Some green algae develop flagella of unequal length but apparently otherwise similar ultrastructure [5, 6]. In contrast, *Chlamydomonas reinhardtii* and other isokont algae have two flagella of equal length and morphology. Nevertheless, the two flagella are distinct in their responses and sensitivity to calcium [7]. These functional differences between the two flagella of a given cell are likely the basis for the stirring movements that underlie directed swimming, for example, during phototaxis, when cells seek light conditions optimal for photosynthesis [8]. Concomitant assembly of distinct cilia on a given cell likely requires selective entry or retention of proteins into the cilia but most details remain to be explored.

**Fig 1:**
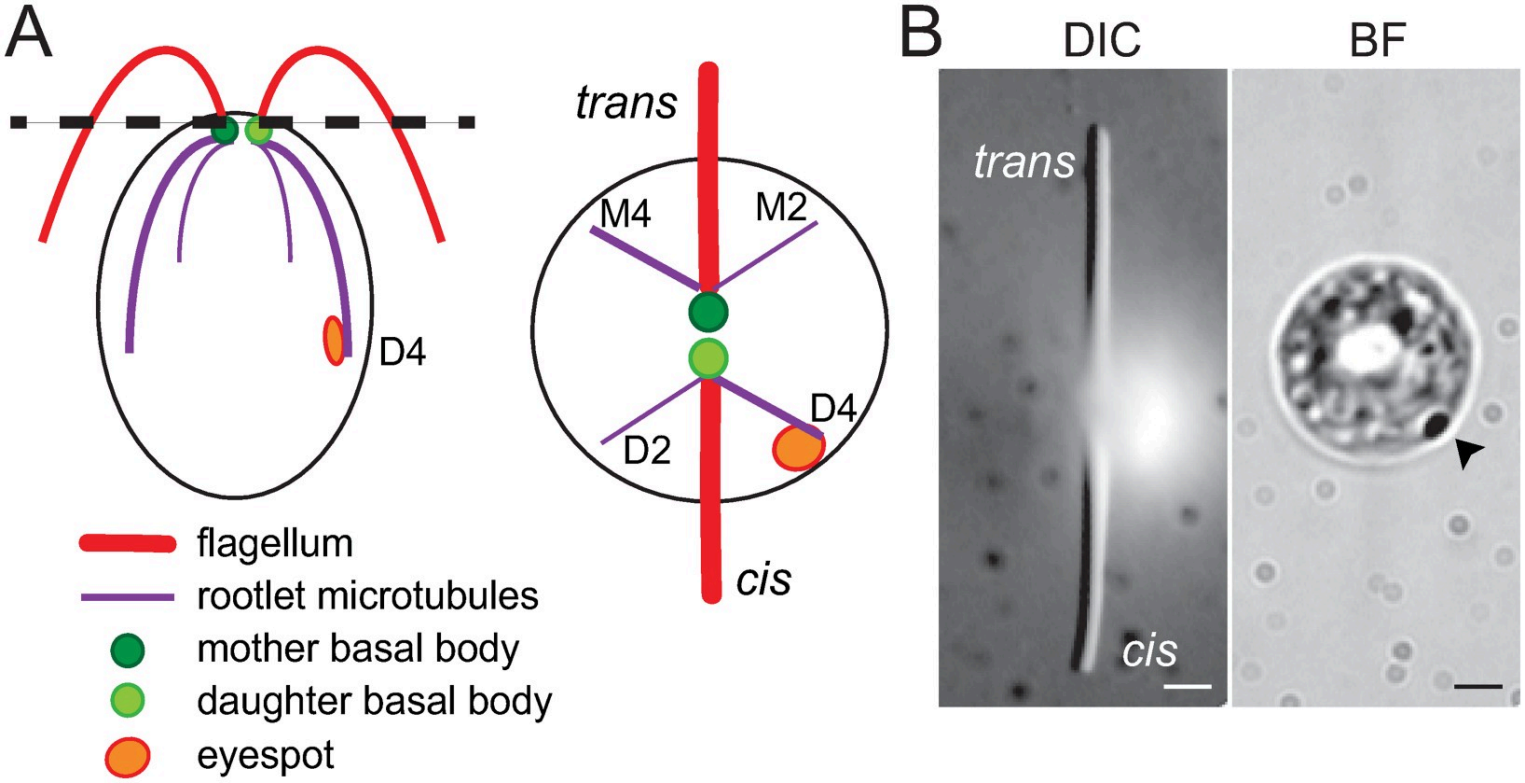
Flagellar asymmetry in *C*. *reinhardtii*. A) Schematic representation of *C*. *reinhardtii* viewed from the side (left) and from the top (right). Flagella (red) extend from the two basal bodies (green), each of which is associated with two microtubular rootlets (purple). The eyespot (orange) is associated with the 4-standed microtubular rootlet (D4) linked to the daughter basal body. Thus, the *cis*-flagellum is closer to the eyespot than the *trans*-flagellum. The rootlets are named to indicate the number of microtubules (2 or 4) and their association with the mother (M) or daughter (D) basal body [12]. (B) Differential interference contrast (DIC) and brightfield (BF) images of a live cell captured at two different focal planes showing the flagella (left) and eyespot (right) indicated with an arrowhead. Bar = 2μm.

The differences between the two flagella of *C*. *reinhardtii* and other flagellates are related to the developmental age of the associated basal bodies [5, 9]. In *C*. *reinhardtii*, the younger basal body organizes the *cis*-flagellum and is connected via a four-stranded microtubular root to the sole eyespot apparatus, which is positioned on one side of the cell body (Fig 1A). The *trans*-flagellum emerges from the older basal body and faces the side opposite of the eyespot (Fig 1B). The differences between the two flagella become even more apparent after mechanical removal of either the *cis* or the *trans*-flagellum: the remaining *cis*-flagellum of such uniflagellate cells beats with the frequency of standard biflagellate cells whereas the beat frequency of a *trans*-uniflagellate cell is much higher [10, 11]. Thus, the motility of the two flagella, likely synchronized by hydrodynamic coupling when paired, is intrinsically distinct indicating differences in their composition and structure. However, biochemical differences distinguishing the *cis* and *trans*-flagellum of *C*. *reinhardtii* have not yet been identified.

Non-phototactic mutants of *C*. *reinhardtii* that lack calcium-dependent differences in flagellar dominance could be instrumental in identifying protein signatures specific for the *cis*- and *trans*-flagellum [8, 13, 14]. These include *ptx1*, which is uncharacterized at the molecular level, and *bbs* mutants, which are defective in the BBSome, a conserved octameric protein complex that moves via intraflagellar transport (IFT) through flagella [15–17]. In *C*. *reinhardtii*, the BBSome is required to remove a subset of membrane-associated proteins from flagella and the abnormal accumulation of the BBSome cargo phospholipase D (PLD) in *bbs* mutant cilia is likely causative for the loss of phototaxis [18]. However, additional biochemical defects of *bbs* flagella have been identified and we show here that BBSome loss affects the distribution of carbonic anhydrase 6 (CAH6) in flagella [19].

Carbonic anhydrases, 12 of which are predicted to be encoded in the *C*. *reinhardtii* genome [20], reversibly catalyze the conversion of CO_2_ and H_2_O into bicarbonate or carbonic acid. Aside from light, carbon dioxide (CO_2_) is the other rate-limiting substrate of photosynthesis in the low CO_2_ environments experienced by aquatic algae. CAH activity is critical to capture inorganic carbon and concentrate it near ribulose-1,5-biphosphate carboxylase-oxygenase (Rubisco) in the plastid, for assimilation in a pathway referred to as the carbon concentrating mechanism (CCM) [21]. Interestingly, CAH6 is located in the flagella of *C*. *reinhardtii* but its role in flagella and possible contribution to the CCM remain unknown [19, 22].

Here, we show that CAH6 fused to mNeonGreen (mNG) is preferentially localized to the *trans*-flagellum, making it the first biochemical marker that distinguishes the *cis* and *trans*-flagellum of *C*. *reinhardtii*. Interestingly, the asymmetric flagellar distribution of CAH6 is lost in *bbs* mutants. A *cah6* insertional mutant displayed normal cell motility and tactic cell behaviors including phototaxis and chemotaxis for bicarbonate phototaxis, suggesting that CAH6 is not linked to flagellar steering movements. In vivo analyses indicate that *bbs* mutants fail to prevent CAH6 from entering the *cis*-flagella revealing a novel role for the *C*. *reinhardtii* BBSome in controlling protein entry into flagella and establishing *cis*/*trans*-flagellar asymmetry.

## Materials and methods

### Strains and culture conditions

*C*. *reinhardtii* was maintained in modified M medium at 24°C with a light/dark cycle of 14:10 h (www.chlamycollection.org/methods/media-recipes/minimal-or-m-medium-and-derivatives-sager-granick/). For gamete generation, cells were resuspended in M without nitrogen (M-N), and aerated for 15–18 h in constant light. Then, cells were incubated in 20% M-N with 10 mM HEPES, pH 7, for 1 h in constant light with agitation. The following strains were used in this study: *cah6*, *cah6* CAH6-mNG, *bbs1 cah6* CAH6-mNG, *bbs4-1* and *g1* as wild-type control strain. All other strains have been previously described and are available at the Chlamydomonas Resource Center (www.chlamycollection.org/).

### Transgenic strain generation

To express mNG-tagged CAH6, the coding portions of the gene were amplified via PCR using strain CC-620 genomic DNA as a template; the PCR fragment was inserted into the previously described ipBR vector, placing *CAH6* downstream of the *HSP70A-rbcS2* fusion promoter and upstream of the mNG gene followed by the 2A sequence, the selectable marker *BLE*, and the *rbcS* 3’ terminator sequence (Fig 3A) [18, 23, 24]. For transformation, the plasmid was linearized with KpnI, gel-purified and transformed into *C*. *reinhardtii* by electroporation. Transformants were selected on Tris-acetate-phosphate (TAP) plates containing 5 to 10 μg/mL zeocin (Invitrogen), and clones expressing mNG-tagged protein were identified by TIRF microscopy (https://www.chlamycollection.org/methods/media-recipes/tap-and-tris-minimal/). Mating was used to introduce CAH6-mNG into other strains.

### Flagellar isolation and western blot

To isolate flagella for protein analysis, we followed a protocol described by [25]. In brief, cells were concentrated and washed in 10 mM HEPES, pH 7.4, resuspended in HMS at 4°C (10 mM HEPES, 5 mM MgSO_4_, and 4% sucrose) and deflagellated by adding dibucaine to a final concentration of 4.17 mM (Sigma-Aldrich) and repeated pipetting. Flagella were separated from cell bodies by centrifugation, collected from the supernatant by centrifugation, and resuspended in HMEK (30 mM HEPES, 5 mM MgSO_4_, 25 mM KCl, and 0.5 mM ethylene glycol-bis(β-aminoethyl ether)-*N*,*N*,*N*′,*N*′-tetra acetic acid [EGTA]) with protease inhibitor cocktail (Sigma-Aldrich). For Western blotting, SDS–PAGE sample buffer was added to flagella samples and samples were incubated at 85°C for 10 min. To raise a polyclonal antibody to CAH6, the C-terminal peptide encoded by the last exon of *CAH6* was fused downstream of maltose-binding protein (MBP), the recombinant fusion protein was purified using amylose-sepharose and used to immunize rabbits (Pocono Rabbit Farm). For affinity-purification, we used the fusion protein immobilized on nitrocellulose membrane. The following primary antibodies were used: rabbit anti-CAH6 (1:500), mouse anti-IC2 (1:1,000) [26] rabbit anti-BBS4 (1: 500) [17], mouse anti-α-tubulin (1:10,000; Sigma) and mouse anti-IFT81 (1:1,000) [27]. Western blots were developed using anti-mouse and anti-rabbit IgG conjugated to horseradish peroxidase (Invitrogen) and chemiluminescence substrate (Michigan Diagnostics). Images were captured using a BioRad Gel Doc imaging system.

### Flagellar regeneration

For flagellar regeneration, cells in M medium were deflagellated by a pH shock (∼pH 4.3 for ∼45 s), pelleted, and resuspended in fresh M medium. Flagella regrowth was allowed to proceed at room temperature under constant light and agitation. To delay flagellar regeneration, cells were kept on ice until needed.

### *In vivo* microscopy

For in vivo imaging, a Nikon Eclipse Ti-U inverted microscope equipped with a 60×/1.49 numerical aperture (NA) TIRF objective and a through-the-objective TIRF illumination system was used. Excitation light was provided by a 40-mW, 488-nm diode laser (Spectraphysics), and filtered by a Nikon GFP/mCherry TIRF filter cube [28, 29]. The emission was documented at 10 frames/s using an EMCCD camera (Andor iXon X3 DU897) and the Elements software package (Nikon). For photobleaching the entire flagellum, the laser intensity of the 488-nm laser was increased to 10% for 4–10 s. To prepare the observation chamber, 10 μl of cells was placed on a 24 × 60 mm no. 1.5 coverslip previously applied with a ring of petroleum jelly. The cells were allowed to settle for ∼1–10 min, mixed with an equal volume of 10 mM HEPES and 6.25 mM EGTA (pH 7.4) under a 22 × 22 mm no. 1.5 coverslip. The images were analyzed and kymograms and walking averages were generated in FIJI (= ImageJ; National Institutes of Health). Merged images or kymograms were produced using Photoshop and figures were assembled in Illustrator (Adobe).

### FRAP and fluorescence intensity analysis

For FRAP analysis, videos were opened in ImageJ, and the region of interest (ROI) was selected using the rectangle tool. The fluorescence intensity inside the selected region was determined using the “Plot Z-axis Profile” tool, and the data were exported into Excel. The fluorescence intensity in the ROI was corrected for the background fluorescence. The highest intensity value before the bleaching event was set to 100%, and the recovery of fluorescence in percentage of the prebleached value was calculated.

### Membrane inlet mass spectrometry (MIMS)

^18^O-labeled dissolved inorganic carbon (DIC) was added to assay buffer [DIC-free water, 20 mM Bicine, pH 8.0] in the MIMS chamber as previously described by [30]. ^18^O exchange was monitored for ∼10 min before the addition of lysed concentrated cells or isolated flagella to determine the background rate of hydration and dehydration. After addition of cells or flagella, ^18^O exchange was monitored for an additional 10–20 min to determine the acceleration of ^18^O removal by CA. The CA-enhanced rate of CO_2_ hydration (k_cf_) was determined by fitting the ^18^O data to a model as described previously (Silverman 1982, [31]) and then normalized to assay protein concentration and the spontaneous CO_2_ hydration rate (k_uf_) as a measure of CA activity.

### Phototaxis and chemotaxis assays

Population phototaxis assays were performed by placing a cell suspension (∼10^6^ cells per milliliter) into a Petri dish, followed by illumination with light from one side for ∼5 min. Images were taken with a standard digital camera (OM-D; Olympus). For the control, we used *g1* (*nit1*, *agg1*, mt+), a strain selected for strong negative phototaxis [8]. Cells were washed with fresh M-medium before assaying phototaxis.

Population chemotaxis assays were performed by placing a cell suspension (∼10^6^ cells per milliliter) into a Petri dish, followed by the addition of an agar pellet containing 5% sodium bicarbonate for ∼20 min in complete darkness to prevent potential interference from light [32]. Agar pellets containing 20% tryptone were used as a positive control [32] and agar pellets prepared with medium were used as a negative control. Images were taken with a standard digital camera. Cells were washed with fresh M-medium prior to the chemotaxis assays.

## Results

### Identification of a *cah6* null mutant

**Fig 2:**
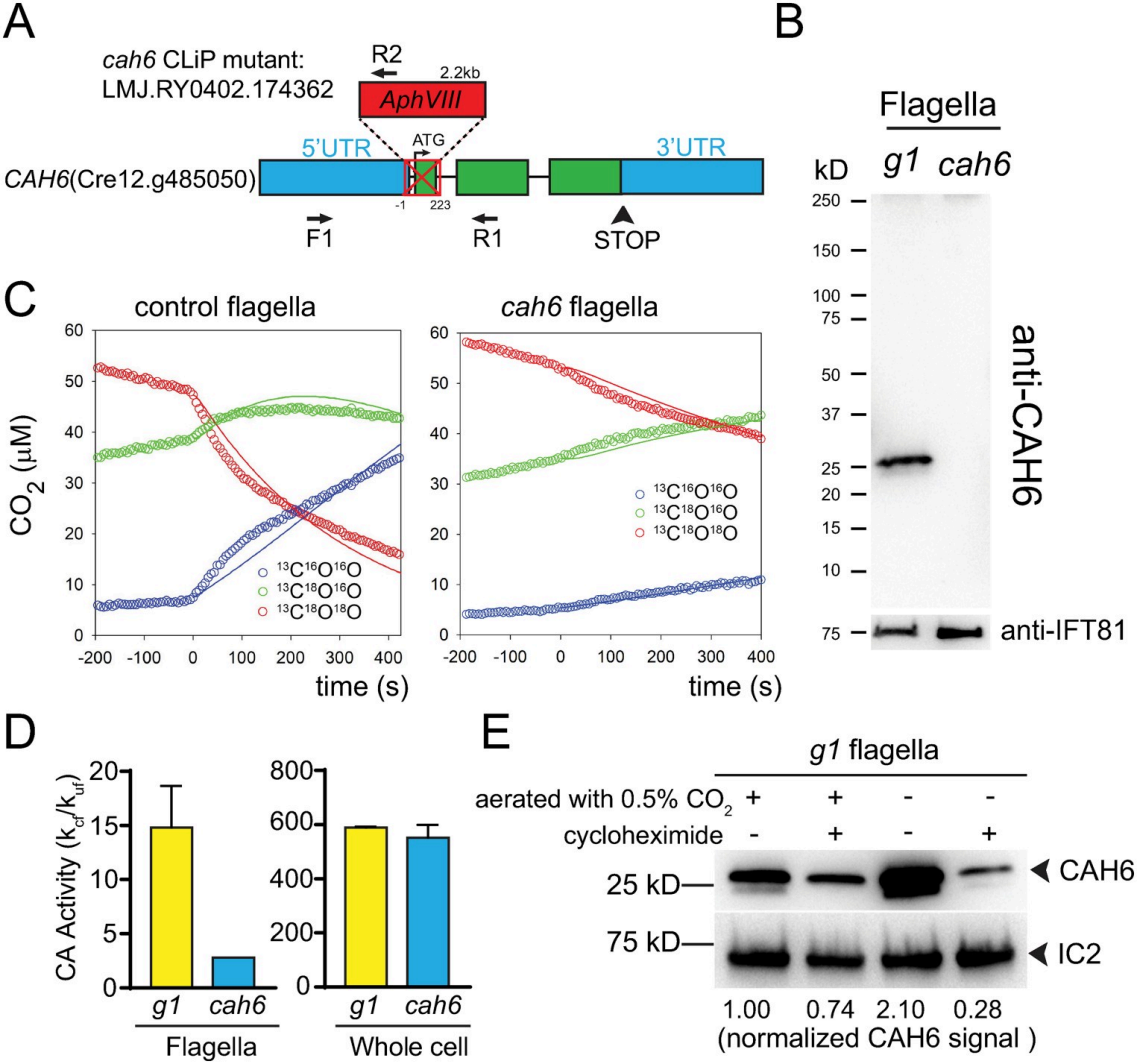
Carbonic anhydrase 6 is active in flagella. A) Schematic presentation of the *CAH6* gene and the insertion in the *cah6* mutant. The insertion of the *APHVIII* cassette caused a deletion (indicated by a red crossed rectangle) of 224 bp encompassing the start codon and first exon of CAH6. The positions of the primers (F1, R1, and R2) used to track the mutation by PCR are indicated (see S1A Fig). B) Western blot analysis of isolated flagella from wild-type (*g1)* and the *cah6* mutant were probed with anti-CAH6. Antibodies to the IFT particle protein IFT81 were used to control for equal loading. C) ^18^O-exhange measurements (open circles) and models (solid lines) for control (*g1)* and *cah6* flagella. Three ^13^CO_2_ isotopologues are tracked and analyzed: ^13^C^16^O^16^O (blue circles), ^13^C^18^O^16^O (green circles) and ^13^C^18^O^18^O (red circles). After determining the background rate of exchange (before time 0), isolated flagella from the strain indicated were added at time 0 and the exchange rate was measured for 400 seconds. CA activity is indicated by the accelerated conversion of ^13^C^18^O^18^O into ^13^C^18^O^16^O and ultimately ^13^C^16^O^16^O. The best fit line (k_cf_) of the model after addition of isolated flagella is shown. D) Flagellar and whole cell CA activity (k_cf_/k_uf_) of *g1* and *cah6*; the values were normalized for the protein concentration (mg/mL) in the respective samples and are based on repeat measurements of the same samples (n = 1). E) Western blot analysis for CAH6 in wild-type (*g1*) flagella. Cells were treated for 24 hours prior to the flagellar isolation as follows: The cultures were either aerated with high CO_2_ (0.5%) or maintained without aeration both in the presence (+) or absence (-) of cycloheximide. Anti-IC2 was used as a loading control.

From the *C*. *reinhardtii* CLiP mutant collection, we obtained strain LMJ.RY0402.174362 that carries an insertion in the *CAH6* gene causing a 224bp deletion encompassing the first intron and the first exon including the start codon (Fig 2A). The insertion in the *cah6*^CLIP^ mutant was verified by PCR, which shows the expected ~2.8-kb size of the amplicon using primers flanking the insertion and a product of ~700bp when a primer specific for the inserted cassette was combined with a *CAH6* gene-specific primer (S1A Fig) [33]. To eliminate possible second site mutations in *cah6*^CLIP^, we outcrossed the *cah6*^CLIP^ mutant to the wild-type strain *g1* resulting in strain *cah6*, which was used for all subsequent experiments. Western blot analysis of flagella isolated from control cells (*g1*) using a polyclonal antibody raised against the peptide encoded by the last exon of *C*. *reinhardtii* CAH6 identified a band of ~25kDa, which is close to the predicted size of CAH6 (28 kDa) (Fig 2B). The corresponding band was absent in flagellar samples of *cah6* strain indicating that the mutant lacks CAH6 (Fig 2B). For reasons unknown, the antibody failed to detect CAH6 in western blot analyses of the more complex whole cell and cell body samples (S1B Fig). Our data confirm that CAH6 is present in *C*. *reinhardtii* flagella [19, 22, 34].

### Carbonic anhydrase 6 is active in flagella

Based on the presence of 21 of 23 highly conserved residues, *C*. *reinhardtii* CAH6 belongs to the β-family of carbonic anhydrases (CAs), which is present in prokaryotes and the plastids of higher plants and algae (Fig 1C and 1D) [35]. In addition, the N-terminal domain of CAH6 is predicted to encompass a chloroplast transit peptide for import into the plastid (S1C and S1D Fig). Thus, it is possible that some CAH6 is imported into the plastid [36]. However, CAH6 was identified in the *C*. *reinhardtii* flagellar proteome, our western blot analyses suggest that only a small pool of CAH6 is present in the cell body and a CAH6-YFP signal was not detected in the plastid [19, 22, 34]. Further, the N-terminal region of CAH6 is predicted to be dual acylated (S1D Fig), a known flagellar localization signal that likely is also responsible for attaching the protein to the flagellar membrane [37]. The data suggest that CAH6 is predominately a flagellar protein.

To analyze the role of flagellar CAH6, we measured carbonic anhydrase (CA) activity in flagella isolated from control and *cah6* using membrane inlet mass spectrometry (MIMS) based on ^18^O removal from ^13^CO_2_ isotopologues (S1E Fig) [38]. Compared to the spontaneous conversion observed prior to addition of the sample (T-200 to T0), control flagella significantly accelerated the conversion of ^13^C^18^O^18^O to ^13^C^16^O^16^O with a CA activity of 14.81 k_cf_/k_uf_ per mg protein (Fig 2C and S1 Table). In contrast, CA activity was more than 5-fold lower in the *cah6* mutant flagella (Fig 2D). In whole cell samples of both strains, we measured an approximately equal CA activity (Fig 2D). All other characterized CAs of *C*. *reinhardtii* are located in the cell body and in agreement CA activity in whole cell samples was about 40-fold higher than that in wild-type flagella (Fig 2D) [20]. Likely, the residual CA activity in *cah6* flagella results from a contamination of the flagellar sample with cell body material. Since CAH6 is the only known CA in *C*. *reinhardtii* flagella [20, 22], we conclude that CAH6 is active in flagella.

In *C*. *reinhardtii*, the expression of CAs and other proteins associated with the carbon concentrating mechanism (CCM) is upregulated in response to limited supply of inorganic carbon in the environment [39–41]. To test if CAH6 levels in flagella adjust with changes in the environmental CO_2_ levels, we maintained *g1* cells aerated with air containing low (0.04%) or high (0.5%) levels of CO_2_ and without aeration for 24 hours and analyzed the flagella with anti-CAH6. In comparison to the high CO_2_ sample, the amount of CAH6 in flagella was increased approximately 2-fold when cells were treated with air (~0.04% CO_2_) and more than 10-fold in cells maintained without aeration (Fig 2E, S2E Fig). To test whether a low environmental CO_2_ concentration induces *de novo* synthesis of CAH6 or its redistribution from the cell body into the flagella, wild-type cells were incubated at high CO_2_ (0.5%) and without aeration for 24 hours both in the absence or the presence of cycloheximide, an inhibitor of proteins synthesis. An increase of CAH6 in flagella as it occurred in controls without cycloheximide in response to the lack of aeration, was not observed in the cycloheximide treated sample (Fig 2E). Instead, cycloheximide treatment reduced the amount of CAH6 in the flagella probably because the block of protein synthesis prevented the replacement of CAH6 lost by turnover. Thus, the increased presence of CAH6 in flagella at low environmental CO_2_ likely results from an increased expression and *de novo* synthesis of CAH6. The data suggest that CAH6 is active in flagella and that CAH6 expression is significantly up-regulated under environmental CO_2_ limitation as described for other CCM proteins. The *cah6* mutant, however, lacks an apparent phenotype as it developed flagella of normal length (S2A Fig), swam with wild-type velocity (S2B Fig), displayed positive chemotaxis for bicarbonate (S2F and S2G Fig), negative phototaxis (S2D Fig) and grew at a similar rate as the *g1* control strain (S2C Fig).

### The BBSome ensures a preferential localization of CAH6-mNG to the *trans*-flagellum

**Fig 3:**
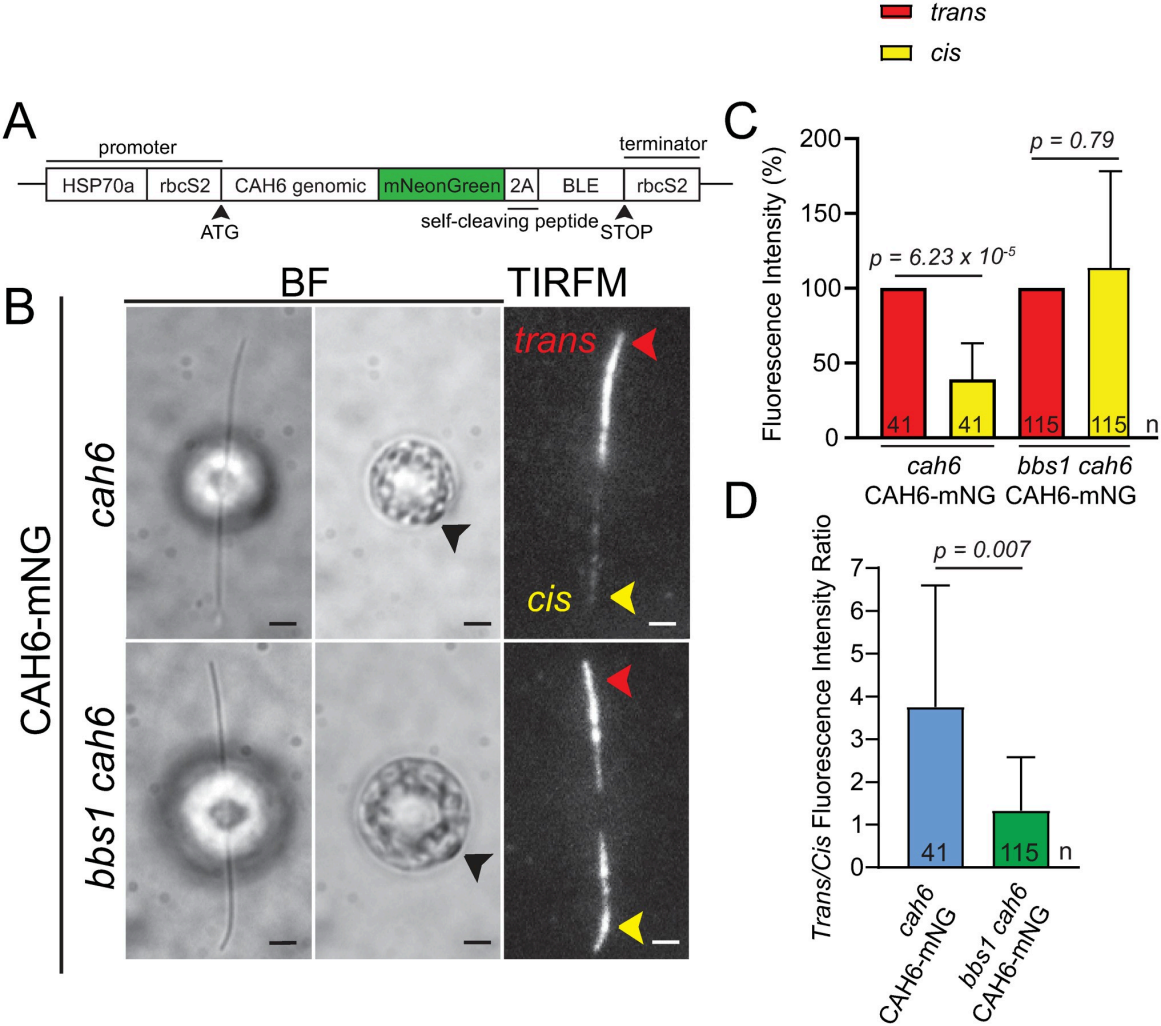
The asymmetric localization of CAH6-mNG in flagella is lost in the *bbs1* mutant. A) Schematic representation of the CAH6-mNG expression vector. The sequence for mNG was inserted at the 3’ end of the genomic *CAH6* coding region. B) Brightfield (BF) images of the flagella (left) and the eyespot (right, indicated with black arrowheads) and the corresponding TIRF images of live *cah6* CAH6-mNG and *bbs1 cah6* CAH6-mNG cells. The *trans*-flagella are indicated with red arrowheads and the *cis*-flagella with yellow arrowheads. Bar = 2μm. C) Histograms of the fluorescence intensity of CAH6-mNG in the *trans*- and *cis*-flagella of the *cah6* CAH6-mNG rescue strain and the *bbs1 cah6* CAH6-mNG strain. For each cell, the fluorescence intensity in its *trans*-flagellum was set to 100% and the fluorescence intensity of the corresponding *cis*-flagellum is displayed as % of that of the *trans*-flagellum. One cell, lacking detectable CAH6-mNG in the *cis*-flagellum and thus having an outlier *trans/cis*-ration of 158, was ignored for the statistical analyses. Error bars indicate the standard deviation. n = number of cells analyzed. D) Histogram of the ratio of the fluorescence intensity of CAH6-mNG between the *trans*- and the *cis*-flagellum in the *cah6* CAH6-mNG and the *bbs1 cah6* CAH6-mNG strain; shown is the mean of the ratios determined for the individual trans/cis pairs of individual cells. Error bars indicate the standard deviation and n the number of flagellar pairs analyzed. See S3C Fig for the individual data points.

When fixed with formaldehyde or -20°C methanol, the flagella of both control and *cah6* mutant cells were weakly stained revealing that the antibody is unsuited for immunocytochemistry (not shown); we obtained similar results with previously described antibodies to *C*. *reinhardtii* CAH6 [36]. To gain insights into the distribution of CAH6, we fused mNeonGreen to the C-terminus of CAH6 and expressed the fusion protein in the *cah6* mutant using a bicistronic vector encompassing the auto-cleavable 2A sequence and the *BLE* gene conferring resistance to zeocin for selection (Fig 3A) [18, 23]. C-terminal tagging was used because the N-terminal region of CAH6 is predicted to be co-translationally myristoylated and post-translationally palmitoylated, modifications that likely ensure the reported association with the ciliary membrane [42]. Potential caveats of this approach include the loss of the endogenous transcriptional regulation of CAH6 and interference of the C-terminal mNG tag with the subcellular targeting or function of CAH6. In western blots of isolated flagella, anti-CAH6 identified a band of ~60 kD representing the CAH6-mNG fusion protein (shown in S3B Fig for a *bbs1 cah6* CAH6-mNG strain).

Surprisingly, in vivo imaging revealed that CAH6-mNG is unequally distributed between the two flagella of a given cell (Fig 3B, S3A Fig). Using the eyespot as a positional marker, we determined that CAH6-mNG preferentially localizes to the *trans*-flagellum (Fig 3B and 3C and S3A Fig). The average fluorescence intensity of CAH6-mNG in the *trans*-flagellum of a given cell exceeded that of the corresponding *cis*-flagellum approximately 4-fold (Fig 3D and, for individual data points, S3C Fig). To our knowledge, CAH6-mNG is the first protein identified to preferentially localize to one of the two flagella of *C*. *reinhardtii*.

We previously reported that the amount of CAH6 in *bbs4* flagella progressively decreases over several hours after flagellar regeneration suggesting a role of the BBSome in the flagellar maintenance of CAH6 [19]. To investigate the distribution of CAH6-mNG in the absence of intact BBSomes, we generated a *bbs1 cah6* CAH6-mNG double mutant by mating. Remarkably, the preferential localization of CAH6-mNG to the *trans*-flagellum is lost in the *bbs1* background and more or less similar amounts of the protein were observed in the *cis*- and *trans*-flagellum of a given cell (Fig 3B, 3C and 3D; S1 Movie). The data indicate that intact BBSomes are required to establish the asymmetric distribution of CAH6-mNG in flagella.

### CAH6-mNG *cis/trans*-asymmetry is established early during flagellar regeneration and maintained dynamically

**Fig 4:**
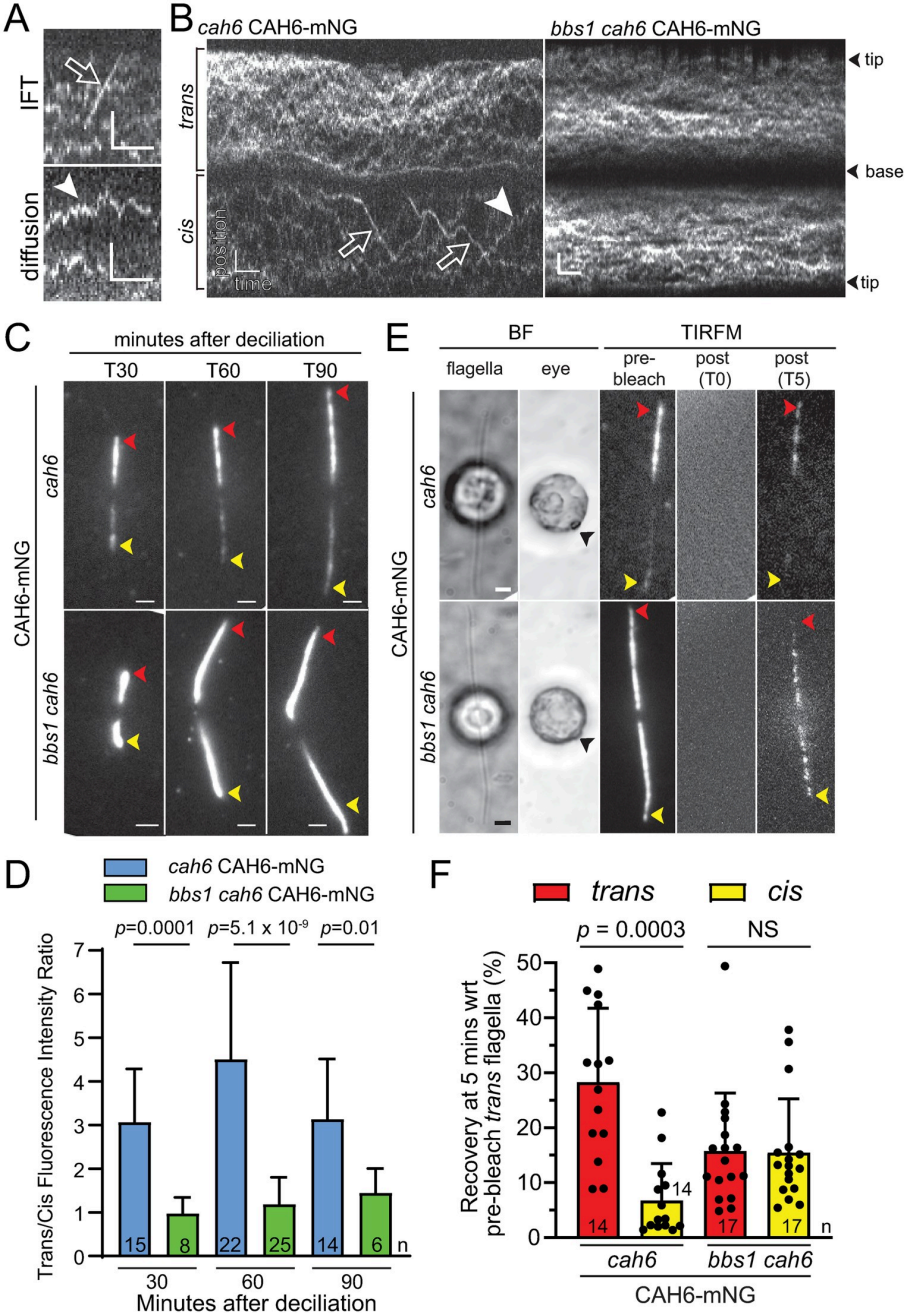
The asymmetric distribution of CAH6-mNG is established early during ciliary assembly and restored after photobleaching. A) Partial kymograms showing IFT (open arrow) and diffusion (white arrowhead) of CAH6-mNG. Scale bars: 2 s and 2 μm. B) Kymograms showing the movements of CAH6-mNG in full-length *cah6* CAH6-mNG and *bbs1 cah6* CAH6-mNG flagella. CAH6-mNG moved mostly by diffusion (white arrowhead) but occasional transport by IFT (open arrows) was observed as well. Scale bars: 2 s and 2 μm. C, D) Analysis of CAH6-mNG in regenerating flagella. C) Still images showing the distribution of CAH6-mNG during flagella regeneration of *cah6* CAH6-mNG and *bbs1 cah6* CAH6-mNG cells. TIRFM images of different live cells were obtained at 30 minutes (T30), 60 minutes (T60) and 90 minutes (T90) after deciliation by a pH shock. Bar = 2μm. D) Quantification of the ratios of CAH6-mNG fluorescence intensity between regenerating *trans*- and *cis*-flagella at 30, 60 and 90 minutes after deciliation. The error bars, indicating standard deviation, and the number of cells analyzed (n) are indicated. E, F) FRAP analysis of CAH6-mNG in full-length flagella. E) Brightfield (BF) of *cah6* CAH6-mNG and *bbs1 cah6* CAH6-mNG cells showing optical section through the flagella and the eyespot (indicated with a black arrow). The corresponding TIRF images show the flagella before (pre), immediately after (T0) and 5 minutes after (T5) the photobleaching step. While partial, the recovery pattern indicated that CAH6-mNG *cis/trans*-asymmetry in wild-type cells is maintained dynamically, i.e., involves continuous entry and exit of CAH6-mNG from flagella. Bar = 2μm. F) Histograms with individual data points comparing recovery of CAH6-mNG fluorescence in *trans*- and *cis*-flagella at 5 minutes after photobleaching. Data are presented as percentage of the prebleach intensity of the *trans*-flagellum and were calculated for each flagella pair. The significance, based on a two-tailed *t* test, is indicated. n = number of flagella analyzed.

To determine how the asymmetric flagellar localization of CAH6-mNG is established, we analyzed the dynamics of CAH6-mNG in full-length and re-growing flagella. Live imaging showed that CAH6-mNG moved by a slow random walk in the *cis*- and *trans*-flagella of both the *cah6* CAH6-mNG and *bbs1 cah6* CAH6-mNG strains (Fig 4A and 4B). This movement is reminiscent of that of phospholipase D (PLD)-mNG, which, similar to CAH6, is predicted to be associated to the flagellar membrane by dual acylation [18]. In contrast to PLD, transport of CAH6-mNG by IFT was rare in both the control and the *bbs1* strain (~0.3 and ~0.4 events/flagellum/minute, respectively) and we did not observe a significant difference in the frequency of CAH6-mNG transport between the *cis*- and the *trans*-flagella (Fig 4A and 4B). During flagellar regeneration, the preference of CAH6-mNG for the *trans*-flagellum was already apparent in short flagella (~4μm) of the *cah6* CAH6-mNG strain whereas it was mostly equally distributed in the two short flagella of *bbs1 cah6* CAH6-mNG cells (Fig 4C and 4D). IFT of CAH6-mNG remained scarce during flagellar regeneration. The data indicate that CAH6-mNG *cis/trans*-asymmetry in control cells is established early during flagellar regrowth. CAH6-mNG *cis/trans* asymmetry could be static with a fixed amount of CAH6-mNG deposited preferentially in *trans*-flagellum during assembly and remaining trapped. Alternatively, the asymmetry could be maintained dynamically with a continuous exchange of CAH6-mNG between flagella and the cell body and distinct rates of entry and/or retention between *cis* and *trans*-flagellum. Fluorescence recovery after photobleaching (FRAP) analyses revealed a partial recovery (25% or less depending on flagellar type and strain) of the CAH6-mNG signals within 5 minutes; the recovery occurred asymmetrically in control cells and symmetrically in *bbs1* cells (Fig 4E and 4F). In conclusion, *cis/trans*-asymmetry of CAH6-mNG is maintained dynamically with the protein continuously entering and exiting the flagella at distinct rates.

### The BBSome restricts entry of CAH6-mNG into the *cis*-flagellum

Previously, we showed that the *C*. *reinhardtii* BBSome functions as a cargo adapter exporting proteins like PLD from flagella by mediating their transport on IFT trains [18]. To determine the kinetics of PLD-mNG removal from *bbs* flagella, we used dikaryon rescue experiments fusing control and *bbs1* PLD-mNG gametes and monitored the removal of PLD-mNG from the *bbs1*-derived flagella after cell fusion by TIRF microscopy (S4A Fig). Even at the earliest time points (≤ 30 min after mixing of the gametes), an abnormal accumulation of PLD-mNG in *bbs1*-derived flagella was not observed, indicating that BBSomes provided by the wild-type parent quickly enter the *bbs1*-derived flagella and rapidly remove PLD-mNG (S4A Fig) [19].

**Fig 5:**
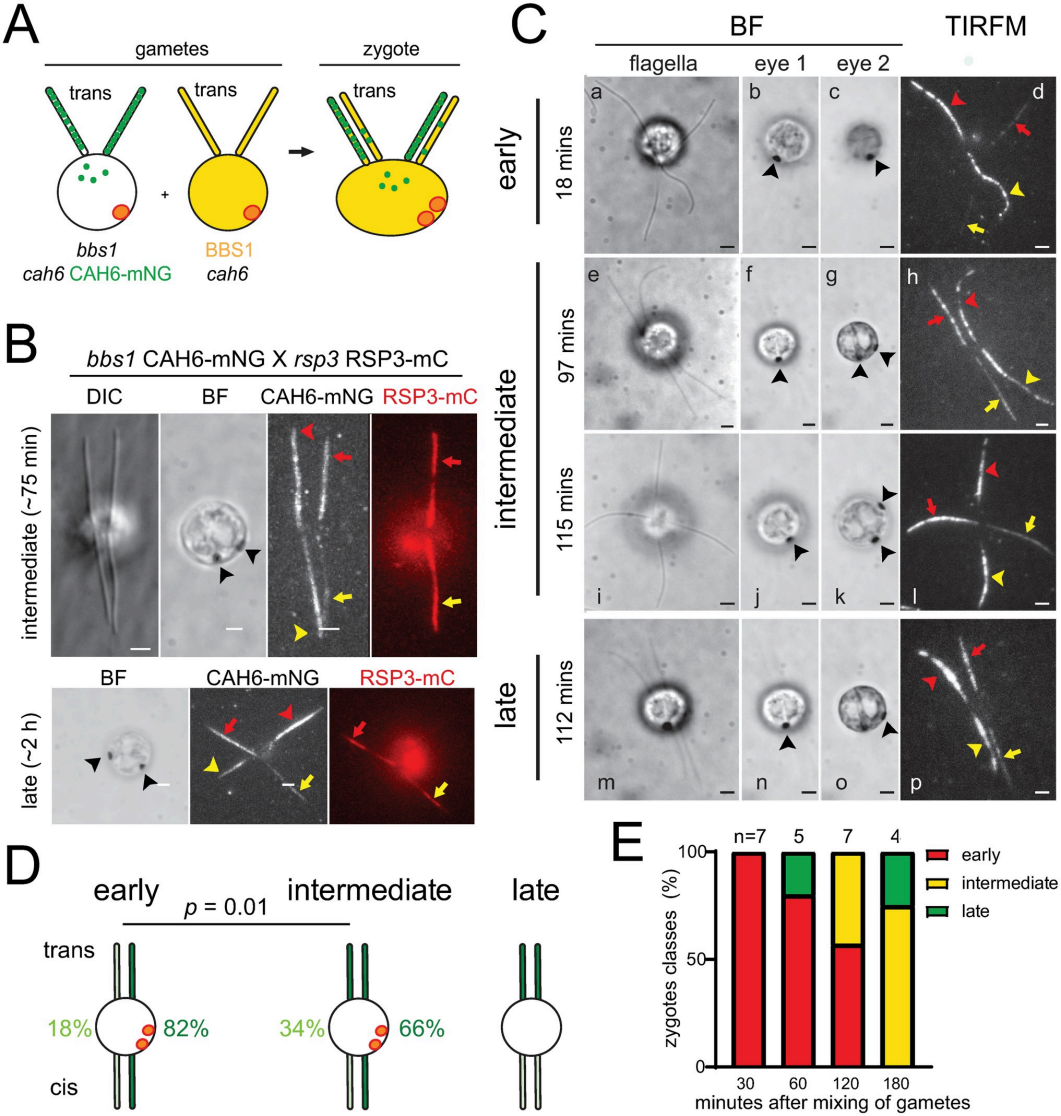
Export of CAH6-mNG from *bbs1*-derived *cis*-flagella is slow. A) Schematic representation of a mating between *bbs1 cah6* gametes expressing CAH6-mNG (green dots) and *cah6* gametes possessing intact BBSomes (indicated by yellow shading). After cell fusion, BBSomes and CAH6-mNG are present in the shared cytoplasm of the zygotes and will enter all four cilia, allowing us to study both the entry of CAH6-mNG into *cah6*-deficient flagella and the BBSome-dependent repair of CAH6-mNG distribution in the *bbs1*-derived flagella. B) Differential interference contrast (DIC), brightfield (BF) and TIRFM images of live zygotes from a mating between *bbs1* CAH6-mNG and a *rsp3* RSP3-mCherry strain; the RSP3-mCherry tag allows us to distinguish *bbs1*- (arrowheads) and wild type-derived flagella (arrows) after cell fusion. Red arrows and arrowheads indicate the *trans*-flagella and yellow arrows and arrowheads indicate the *cis*-flagella. Note that establishing CAH6-mNG *cis/trans* asymmetry in the *rsp3* RSP3-mCherry-derived wild-type cilia precedes correcting the distribution of CAH6-mNG in the *bbs1*-derived flagella. Bar = 2 μm. C) Brightfield (BF) images of different focal planes showing the flagella and the two eyespots (indicated with arrowheads) and the corresponding TIRF image of live *bbs1 cah6* CAH6-mNG × *cah6* zygotes; the time passed since mixing of the gametes is indicated. The *bbs1*-derived flagella are marked by arrowheads (red for *trans* and yellow for *cis*) and the *cah6*-derived flagella are marked correspondingly with arrowheads. Bars = 2 μm. (D) Diagrams of the classification system of zygotes based on the distribution pattern of CAH6-mNG. Early stages possessed one flagellar pair containing ~82% of the total CAH6-mNG signal and a weaker pair accounting for ~18% of the signal. Intermediate stages possessed three flagella with strong signals and one with a weaker signal; accordingly, the signal strength of the stronger cis/trans-pair was reduced to 66% and that of the weaker pair increased to 34% of the total CAH6-mNG fluorescence in a given zygote. Late stages, only a few of which were detected, possessed two flagellar pairs with discernable *cis/trans*-asymmetry in the CAH6-mNG signal. (E) Cumulative bar chart showing the distribution of the three different patterns of flagellar CAH6-mNG distribution in zygotes (see panel D) at different time points.

While IFT of CAH6-mNG was rarely observed, this could potentially be explained by the low abundance of CAH6-mNG in the *cis*-flagellum, i.e., if BBSomes and IFT continuously remove CAH6-mNG from the *cis*-flagellum, the chance to observe an actual transport during the short periods covered by our recordings is low. To expose BBSomes to higher concentrations of CAH6-mNG, we fused control and *bbs1* CAH6-mNG gametes and monitored the removal of CAH6-mNG from the *bbs1*-derived *cis*-flagellum after cell fusion by TIRF microscopy (Fig 5A).

First, we mated a CAH6-mNG control strain to the *cah6* mutant to test if CAH6-mNG *cis/trans*-asymmetry is established in zygotes. The resulting zygotes showed CAH6-mNG in the *trans*-flagellum of each pair indicating that CAH6-mNG *cis/trans*-asymmetry is preserved and established *de novo* in zygotic flagella (S4B Fig). This observation agrees with previous data showing that both eyespots are positioned on the same side of the zygote indicating that cell fusion occurs with the flagellar pairs in a parallel configuration with respect to the *cis/trans*-axis [43]. Next, we mated *bbs1* CAH6-mNG cells to *rsp3* RSP3-mCherry (mC) cells, which enabled us to identify the *bbs1*- and the wild type-derived flagella after formation of the zygotes based on the RSP3-mC signal (Fig 5B). Interestingly, the accumulation of CAH6-mNG in the *rsp3* RSP3-mC-derived *trans*-flagellum preceded the removal of CAH6-mNG from the *cis*-flagellum derived from the *bbs1* parent as indicated by zygotes possessing three flagella with a strong CAH6-mNG signal and one flagellum in a *cis*-position with a weak signal (Fig 5B). The pattern suggests that the repair of *bbs1*-derived flagella is a rather slow process. To avoid a possible interference from endogenous CAH6, we mated the *cah6* mutant to the *bbs1 cah6* CAH6-mNG strain (Fig 5C). In the flagella of the resulting zygotes, CAH6-mNG moved mostly by diffusion and only 2 IFT events were observed during the course of this study, resulting in an average of 0.03 IFT events/minute/flagellum (S4C Fig). Early zygotes (≤ 30 min after mixing of the gametes) possessed strong CAH6-mNG signals in the *bbs1*-derived flagellar pair (containing 85% or more of the total CAH6-mNG signal of a given zygote) and a weakly fluorescent pair derived from the wild type-parent (Fig 5Ca-d, 5D and 5E). A different pattern was prevalent at ~30 minutes or more after mixing of the gametes with many zygotes possessing three flagella with a strong CAH6-mNG signal and one with a weaker signal. The latter was always in a *cis* position and, considering the above observation using the RSP3-mCherry strain, is likely derived from the *cah6* parent (Fig 5Ce-l, 5D and 5E). Only at the later time points (>60 minutes), we observed partial or complete formation of *cis/trans*-asymmetry in both flagellar pairs in a subset of zygotes (n = 2; Fig 5B bottom, Fig 5Cm-p, 5D and 5E). Thus, establishing *cis/trans*-asymmetry of CAH6-mNG in the *cah6*-mutant derived flagella of mosaic zygotes outpaces the removal of CAH6-mNG from the *bbs1*-derived *cis*-flagella. We conclude that the rate of CAH6-mNG export during the repair of the *bbs1 cis*-flagella is substantially slower than the rate of CAH6-mNG entry and accumulation in wild-type or *cah6*-derived *trans*-flagella. This observation is best explained by a role of the BBSome in restricting access of CAH6-mNG specifically to the *cis*-flagellum rather than by BBSome-dependent export of CAH6-mNG from the *cis*-flagellum.

To test if BBSomes can restore the control of CAH6-mNG entry into *bbs*-derived *cis*-flagella of zygotes, we mated the *cah6* CAH6-mNG rescue strain to the *bbs4-1* mutant strain. Even at early time points (≤30 min), the majority of the analyzed zygotes had 2 *trans*-flagella with a strong CAH6-mNG signal and 2 *cis*-flagella with weaker signal (S4D Fig). Thus, the presence of BBSomes ensures that the *bbs4*-derived flagella of such zygotes directly develop the characteristic *cis/trans*-pattern of CAH6-mNG. Apparently, the *de novo* assembly of the CAH6-mNG *cis/trans*-pattern in *bbs*-derived flagella is faster than removal of CAH6-mNG from *bbs*-derived flagella. The data further support the notion that the BBSome restricts the entry of CAH6-mNG into the *cis*-flagellum to establish the *cis/trans*-asymmetry of CAH6-mNG in *C*. *reinhardtii* flagella.

## Discussion

### CAH6-mNG is a biochemical marker for the *C*. *reinhardtii trans*-flagellum

We showed that CAH6-mNG is enriched in the *trans*-flagellum of *C*. *reinhardtii*. Two polyclonal antibodies raised against CAH6 or parts of the protein equally stained the flagella of both control and *cah6* mutant cells. Since western blotting revealed the absence of CAH6 from the mutant flagella, the currently available antibodies to CAH6 are unsuited for immunocytochemistry. Thus, the distribution of the endogenous CAH6 in flagella remains unknown. However, GFP alone and other membrane-associate proteins tagged with mNG distribute equally between the two flagella of a given cell [18]. In an independent study, CAH6-YFP expressed using the PsaD promoter also preferably accumulates in one of the two flagella of *C*. *reinhardtii* control cells [22]. Thus, it is likely that the CAH6 portion of the fusion protein mediates its asymmetric flagellar distribution. Remarkably, the asymmetric flagellar distribution of CAH6-mNG is lost in the non-phototactic *bbs1* mutant indicating that defects in a known flagellar protein transport pathway affect the localization of the fusion protein. Clearly, CAH6-mNG impinges on a cellular pathway that established biochemical differences between the two flagella. Taken together, we consider it likely that CAH6-mNG mimics the distribution of endogenous CAH6.

### CAH6 levels in flagella are regulated by CO_2_ concentration

**Fig 6:**
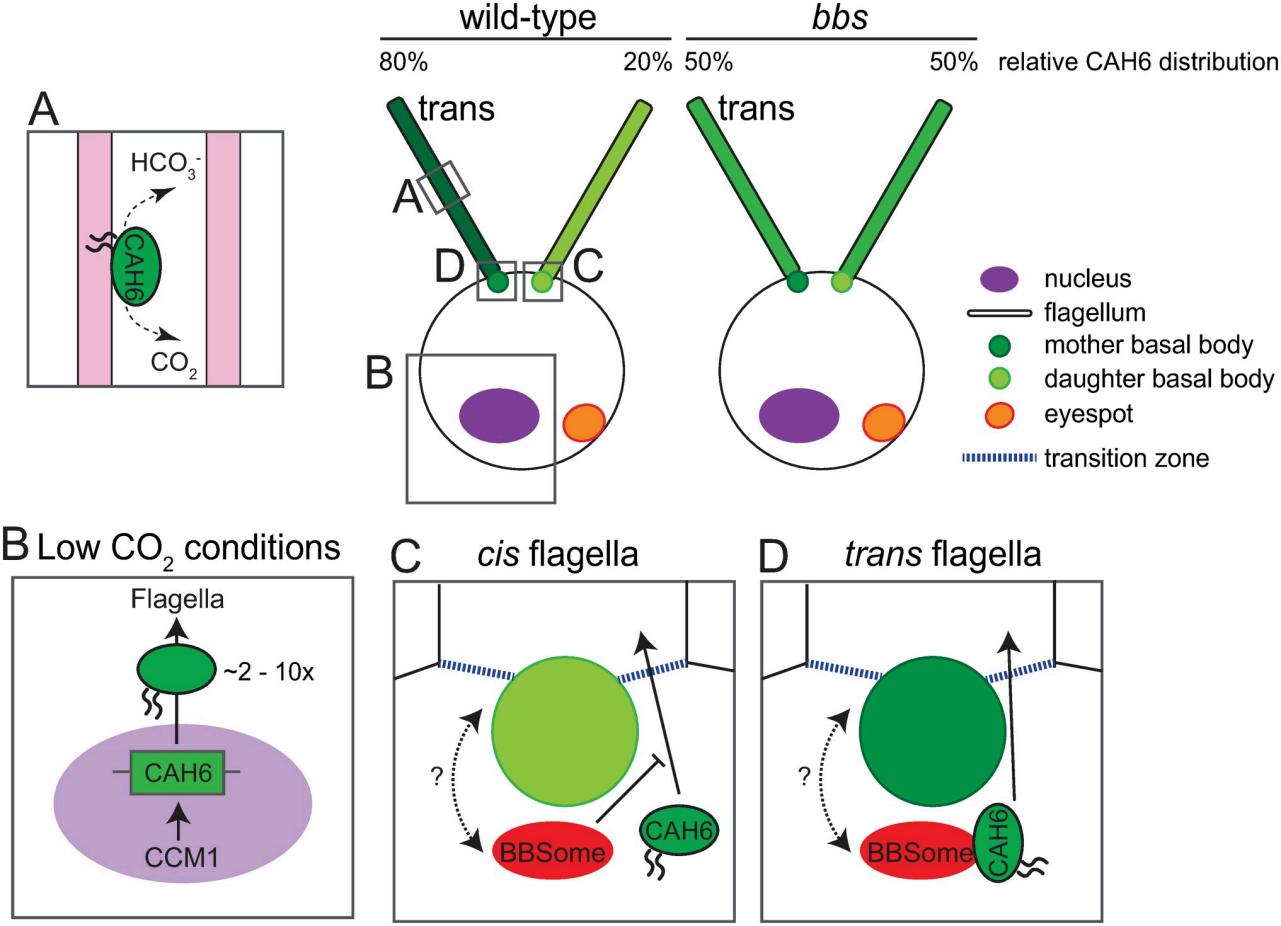
Schematic summary and model of expression regulation and flagellar localization of CAH6-mNG in *C*. *reinhardtii*. A) CAH6 in flagella is actively converting carbon dioxide to bicarbonate and vice versa. CAH6 is predicted to attach to the flagellar membrane (pink) by a dual fatty acid modification (indicated by two wavy lines). B) In CO_2_-limiting conditions, the amount of CAH6 in flagella is upregulated 2- to 10-fold. C, D) The BBSome largely prevents the entry of CAH6-mNG into the *cis*-flagellum. In a hypothetical scenario, the younger basal body could recruit BBSomes to a specific location or activate the BBSome (indicated by the dark red color) to minimize entry of CAH6-mNG into the *cis*-flagellum; during maturation, the older basal body lost its ability to modulate BBSome function and CAH6-mNG will enter the flagella.

CAH6 is apparently active in flagella and its amount in flagella is increased at low levels of CO_2_ due to upregulated expression (Fig 6A and 6B; [44]). An increase in expression under low CO_2_ is typical for most CAs participating in the *C*. *reinhardtii* carbon concentrating mechanism (CCM) [45]. The other known *C*. *reinhardtii* CAs of are located in the periplasm, the cell body cytoplasm and the cell organelles converting CO_2_ to bicarbonate and vice versa to concentrate CO_2_ in the chloroplast. This helps aquatic algae to assimilate CO_2_, which has a low solubility in water. However, endogenous and tagged CAH6 are largely flagellar proteins and it is unclear how CA activity in the flagella could contribute to CO_2_ capture. *C*. *reinhardtii* shows positive chemotaxis for bicarbonate suggesting that its motility is somehow regulated by bicarbonate or CO_2_ [46]. Fungal CAs function as CO_2_ sensors and the regulation of sperm motility in a variety of species involves CAs raising the possibility that CAH6 contributes to CO_2_/bicarbonate chemotaxis of *C*. *reinhardtii* [47, 48]. However, the *cah6* mutant swam with normal velocity, displayed positive chemotaxis for bicarbonate and grew at similar rates as the wild-type control in high and low CO_2_ conditions. The asymmetric flagellar distribution of CAH6-mNG was lost in *C*. *reinhardtii bbs1* mutants, which fail to perform the characteristic steering movements required for phototaxis probably because both flagella of *bbs*-mutants move like wild-type *trans*-flagella [8, 17]. Biochemically, the defect in *bbs* flagella has been linked to the abnormal accumulation of signaling proteins including PLD, which appears to be a negative regulator of phototaxis [18]. However, we consider it unlikely that PLD positively promotes the differential motility of the two flagella because PLD does not display an asymmetric flagellar distribution in either control or *bbs* cells. On the other hand, negative phototaxis was intact in the *cah6* mutant indicating that CAH6 or its asymmetric distribution are not required for light-induced flagellar steering movements. While the role of CAH6 remains enigmatic, it is intriguing that both the asymmetric flagellar distribution of CAH6-mNG and the asymmetric flagellar behavior are lost in the *bbs* mutants. It is possible that other, yet to be identified proteins are also distributed asymmetrically in flagella in a BBSome-dependent manner; such proteins could underly the differential motility of the *cis*- and the *trans*-flagellum required for phototaxis.

### Establishing *cis/trans*-flagellar asymmetry involves the BBSome

Flagellar asymmetry is linked to the developmental differences between the basal bodies that template them. While of similar size and structure, the *trans*- and *cis*-flagellum of *C*. *reinhardtii* are organized by the mother and the daughter basal body, respectively, which are of distinct developmental age and inhabit distinct positions within the cell relative to the single eyespot. Differences between flagella organized by basal bodies of distinct developmental age are apparent in many species and often are more pronounced than in *C*. *reinhardtii* [5, 9]. In mammalian cells, only the mother centriole assembles a primary cilium whereas the daughter basal body requires an additional cell division to become a mother centriole and gain competency to template a flagellum. Heterokont algae possess a long flagellum with mastigonemes attached to the daughter basal body while the mother basal body forms a short smooth flagellum. In *Epipyxis pulchra*, the long flagellum is shortened prior to cell division and loses its mastigonemes as its basal body migrates into the position characteristically occupied by mother basal bodies whereas the short smooth flagellum remains unaltered; each of the two new daughter basal bodies template a long flagellum with mastigonemes [49]. Thus, the basal bodies’ developmental age dictates the structure and composition of the attached flagellum. However, the mechanism by which differences between the flagella of a given cell are established remain unknown. Here, we show that in *C*. *reinhardtii*, the BBSome is required to establish the asymmetric flagellar distribution of CAH6-mNG providing initial mechanistic insight into the process.

### A novel role for the BBSome in limiting protein entry into flagella

In principal, a distinct biochemical composition of the *cis* and *trans*-flagella could result from differences in protein entry or retention. Considering the known role of the BBSome in ciliary protein export, an increased removal of CAH6-mNG specifically from the *cis*-flagellum via the BBS/IFT pathway could establish its asymmetry distribution. However, IFT of CAH6-mNG was rarely observed, the majority of those transports was anterograde, and significant differences in CAH6-mNG IFT frequencies between the *cis* and *trans*-flagella or between control and *bbs1* flagella were not observed. It could be reasoned that in cells with full-length flagella, CAH6-mNG flagellar asymmetry is already established and thus additional transport events will be exceedingly rare. However, flagellar asymmetry of CAH6-mNG is restored at moderate speed after photobleaching indicating that CAH6-mNG continuously enters and exits flagella. Further, the frequency of CAH6-mNG transport remained low during flagellar regeneration when the protein enters cilia *de novo* and its asymmetric distribution develops. In zygotes, IFT remained exceedingly rare during the repair of *bbs1*-derived *cis*-flagella preloaded with CAH6-mNG. Further, restoring CAH6-mNG *cis/trans*-asymmetry in *bbs1*-derived flagella was substantially slower than establishing it *de novo* in cilia initially lacking CAH6-mNG derived from the wild-type, *cah6* or *bbs* parent. While the former involves lowering the flagellar content of CAH6-mNG, the latter can be explained by a BBSome-dependent reduction of CAH6-mNG entry into *cis*-flagella. Thus, BBSome-dependent restriction of CAH6-mNG entry into the cis-flagellum could explain both the rapid development of *cis/trans*-flagellar asymmetry in flagella initially lacking CAH6-mNG and the slow exit of CAH6-mNG form the *bbs1*-derived *cis*-flagella, which likely depends on slow CAH6-mNG turnover. Thus, we conclude that the BBSome specifically limits CAH6-mNG entry into *cis*-flagella of *C*. *reinhardtii*. A role of the BBSome in preventing entry of proteins into cilia could also explain defects in other systems such the abnormal presence of numerous distinct non-ciliary proteins in the outer segment of *bbs* mutant mice [50].

### How could the BBSome establish CAH6-mNG *cis/trans*-asymmetry?

How could the BBSome limit protein entry into flagella, specifically into just one of two flagella in a closely spaced pair? BBS proteins localize to the transition zone, which functions as a ciliary gate, and to the basal body region, which is a platform for the recruitment of ciliary proteins and loading of IFT trains [51–54]. Thus, the BBSome could function as a roadblock preventing CAH6-mNG from approaching the daughter basal body or entering the *cis*-flagellum (Fig 6C and 6D). In mammalian systems, several biochemical differences between the mother and the daughter centriole have been identified [55, 56]. Recent data indicate that cargo binding by the BBSome and probably the passage of the complex through the transition zone require activation of the BBSome likely by the small GTPase Arl6/BBS3 [57, 58]. Similarly, a daughter basal body-specific protein could activate nearby BBSomes enabling them to bind CAH6-mNG and prevent its flow into the attached cis-flagellum. However, in *C*. *reinhardtii*, BBS proteins are present at the base of both flagella and the known structural differences between the two basal bodies are minor, e.g., more cartwheel layers are present in the daughter basal body [17, 59]. In an alternative model, the BBSome could deliver CAH6-mNG selectively to the base of the *trans*-flagellum, which will ensure its preferred entry into the *trans*-flagellum. We previously observed that *bbs*-mutant cilia contain less total endogenous CAH6 than wild-type cilia suggesting a role of the BBSome in the delivery of CAH6 to the flagella [19]. Indeed, the BBSome has been linked to intracellular non-IFT transports such as the dynein-dependent movements of zebrafish melanosomes to the cell center [60, 61]. In *C*. *reinhardtii*, IFT dynein appears to promote entry of SAG-1 into flagella of activated gametes by transporting it along the microtubular roots to the flagellar base [62]. It has been shown that the microtubular rootlets are specialized for specific transports related to *cis/trans*-asymmetry: The MLT1 protein is specifically present along the four-stranded root attached to the daughter basal body of *C*. *reinhardtii* [12]. *Multiple eyespot 1* (*mlt1*) mutants develop additional eyespots in aberrant positions, i.e., on the four-stranded rootlet attached to the *trans*-basal body [63]. MTL1 could specify the four-stranded *cis*-rootlet for the transport of eyespot components ensuring proper positioning of the eyespot [12]. Similarly, rootlets attached to the *trans*-basal body could ensure transport of CAH6-mNG to the base of the *trans*-flagellum in a BBSome-dependent manner whereas BBSome loss diminishes channeling of CAH6-mNG to the *trans*-flagellum causing its aberrant entry into the *cis*-flagellum and an overall reduction of its presence in flagella.

## Supporting information

S1 Table CA activity in whole cell and flagellar samples. The table list the parameters and numerical values of the MIMS experiment for the control (*g1*) and the *cah6* mutant strain.
(DOCX)

S1 Fig CAH6 is active in flagella. A) Agarose gel of PCR products using *cah6* genomic DNA as a template; G-beta primers are used as control. See Fig 2A for the positions of the primers. B) Western blot analysis of whole cells, deflagellated cell bodies (Cell Body), and isolated flagella (Flagella) from wild-type *g1* and *cah6* probed with anti-CAH6. Equivalent amounts of cells and flagella were loaded (i.e., one whole cell, one cell body, and two flagella). Antibodies to the IFT particle protein IFT81 were used to control for equal loading. A part of the same blot is shown in Fig 2B, 2C and 2D) Schematic presentation of CAH6 (C) and amino acid sequence of CAH6 (D). The predicted chloroplast transit peptide (cTP) is indicated in green and the catalytic domain in red. In D, the 23 conserved residues typical for β-type CAs are indicated by red font. Asterisks indicate the two amino acids that are not conserved in CAH6. (E) Schematic representation illustrating the measurement of CA activity.
(TIF)

S2 Fig Phenotypical characterization of the *cah6* mutant. A) Flagellar length of control (*g1*) and the *cah6* strains. The standard deviations and the number of measurements are indicated. B) Mean swimming velocity of wild-type (*g1*) and *cah6* cells in 0.5% CO_2_ and 0.04% CO_2_ (air). C) Liquid growth curves for wild-type *g1* (red circles) and *cah6* (black squares) in 5% CO_2_, 0.04% CO_2_ (air) and <0.04% CO_2_. D) Phototaxis assay of control (*g1*) and *cah6*. The time of light exposure (5 mins) and direction of the light (arrowheads) are indicated. E) Western blot analysis of flagella for CAH6 isolated from wild-type (*g1*) cells aerated for 24 hours under high (0.5%), low (0.04%) and no aeration with CO_2_ conditions. Anti-IC2 was used as a loading control. F) Schematic presentation of the chemotaxis assay. Cells in M-medium were placed into a small Petri dish or 6-well cell culture plate and agar plaques (A, B) containing either 5% sodium bicarbonate or 20% tryptone (A) or no attractant (B) were added. G) Chemotaxis assay showing *g1*, *cah6* and *bbs4-1* before (T0) and after a 20-minute incubation in the dark (T20). Accumulated cells are indicated by the arrows. The following results were noted in repeat experiments (positive chemotaxis/no chemotaxis): *g1* aerated with CO_2_ (0/9), *g1* without aeration (5/5), *cah6* (4/3), *bbs4-1* (1/2), and *bbs1* (2/1).
(TIF)

S3 Fig CAH6-mNG is preferentially localized to the *trans*-flagella in wild-type cells. A) Brightfield (BF) and TIRFM images of live *g1* CAH6-mNG cells. The two focal planes show flagella and eyespot (indicated with black arrowheads). *Trans*-flagella are indicated with red arrowheads and *cis*-flagella with yellow arrowheads. Bar = 2μm. B) Western blot analysis of isolated flagella (Flagella) from wild-type *g1*, *cah6* and *bbs1 cah6* CAH6-mNG were probed with anti-CAH6 and anti-BBS4. Antibodies to acetylated tubulin were used as a loading control. C) Scatter plot showing the ratios between the *trans*- and *cis*-flagellum of control and bbs1 cells; see Fig 3D for presentation as a histogram.
(TIF)

S4 Fig BBSome dependent restoration of PLD-mNG and CAH6-mNG distribution in flagella. A) Brightfield (BF) and TIRFM images of a *bbs1* PLD-mNG gametes and live dikaryons from a matings between *bbs1* PLD-mNG and control *(g1)* or *bbs4*. In *bbs1* PLD-mNG x *bbs4* zygotes, both parental strains lack intact BBSomes and BBSomes need to be assembled *de novo* after cell fusion delaying the export of PLD-mNG. Indeed, an abnormal presence of PLD-mNG was observed in two of the four flagella in a subset of the zygotes analyzed at ≤ 30 min whereas PLD-mNG was essentially absent from all four flagella at the later time points (>30 min). Bar = 2 μm. B) Brightfield (BF) and TIRFM images of a live dikaryon from a mating between *g1* CAH6-mNG and *cah6* gametes. *Trans*-flagella are indicated with red arrowheads and *cis*-flagella with yellow arrowheads. Bar = 2 μm. C) Kymograms showing CAH6-mNG dynamics in the flagella of *cah6* × *bbs1 cah6* CAH6-mNG zygotes. Open arrows indicate transport of CAH6-mNG via anterograde IFT. D) Brightfield (BF) and TIRFM images of live dikaryons from a mating between *cah6* CAH6-mNG and *bbs4* gametes. The two focal planes show the flagella and the eyespot (indicated with black arrowheads). *Trans*-flagella are indicated with red arrowheads and *cis*-flagella with yellow arrowheads. Bar = 2 μm.
(TIF)

S1 Movie CAH6-mNG in control and *bbs1* flagella. TIRF localization of CAH6-mNG in *cah6* CAH6-mNG (left) and *bbs1 cah6* CAH6-mNG (right) flagella. The beginning of the video was recorded in brightfield mode and shows first the eyespot (eye) and then moves to the focal plane of the flagella before switching to TIRF illumination. The *cis*- and *trans*-flagella are indicated. Images were acquired at 10 fps, and playback is set at 20 fps (2× speed). The timer counts seconds.
(AVI)

S1 Raw images (DOCX)

## Abbreviations

BBS: Bardet-Biedl syndrome
CA: carbonic anhydrase
CAH6: carbonic anhydrase 6
CCM: carbon concentrating mechanism
FRAP: fluorescence recovery after photobleaching
IFT: intraflagellar transport
mC: mCherry
MIMS: membrane inlet mass spectrometry
MLT: multiple-eyespot
mNG: mNeonGreen
PLD: phospholipase D
Rubisco: ribulose-1,5-biphosphate carboxylase-oxygenase
TIRFM: Total Internal Reflection Fluorescence Microscopy
