# Supplementary material for: The BBSome restricts entry of tagged carbonic anhydrase 6 into the cis-flagellum of Chlamydomonas reinhardtii
Source: PLoS One. 2020 Oct 29;15(10):e0240887. doi: 10.1371/journal.pone.0240887 (PMC7595284; doi:10.1371/journal.pone.0240887)
Supplement: S1 Table — The table list the parameters and numerical values of the MIMS experiment for the control (g1) and the cah6 mutant strain. (DOCX) [file pone.0240887.s001.docx]

*S1 Table) CA activity in whole cell and flagellar samples*

| **Symbol** | **Definition** | **Flagella** | | **Whole Cell** | | **Units** |
| --- | --- | --- | --- | --- | --- | --- |
|  |  | *g1* | *cah6* | *g1* | *cah6* |  |
| k_uf_ | Background CO_2_ hydration, HCO_3_^-^ dehydration rate constants | 0.03 | 0.0265 | 0.03 | 0.03 | s^-1^ |
| k_cf_ | Carbonic anhydrase catalyzed CO_2_ hydration, HCO_3_^-^ dehydration rate constants | 0.14 | 0.0157 | 1.98 | 1.215 | s^-1^ |
| mg/mL | Protein concentration used to normalize CA activity | 0.328125 | 0.21125 | 0.09765 | 0.064 | mg/mL |

The table list the parameters and numerical values of the MIMS experiment for the control (*g1*) and the *cah6* mutant strain.
