# Supplementary figures and images for: The BBSome restricts entry of tagged carbonic anhydrase 6 into the cis-flagellum of Chlamydomonas reinhardtii
Source: PLoS One. 2020 Oct 29;15(10):e0240887. doi: 10.1371/journal.pone.0240887 (PMC7595284; doi:10.1371/journal.pone.0240887)

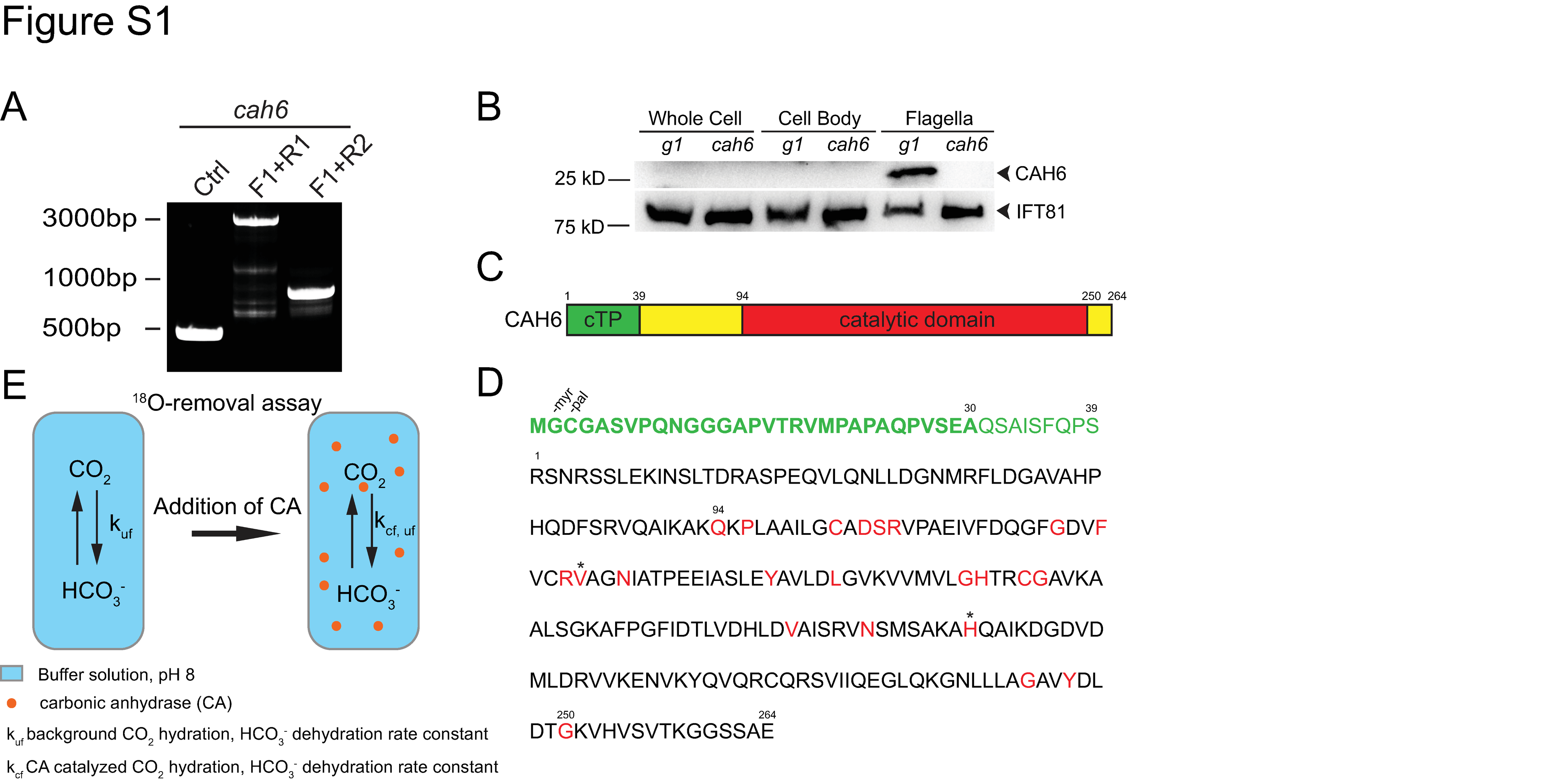

Supplement: S1 Fig — A) Agarose gel of PCR products using cah6 genomic DNA as a template; G-beta primers are used as control. See Fig 2A for the positions of the primers. B) Western blot analysis of whole cells, deflagellated cell bodies (Cell Body), and isolated flagella (Flagella) from wild-type g1 and cah6 probed with anti-CAH6. Equivalent amounts of cells and flagella were loaded (i.e., one whole cell, one cell body, and two flagella). Antibodies to the IFT particle protein IFT81 were used to control for equal loading. A part of the same blot is shown in Fig 2B, 2C and 2D) Schematic presentation of CAH6 (C) and amino acid sequence of CAH6 (D). The predicted chloroplast transit peptide (cTP) is indicated in green and the catalytic domain in red. In D, the 23 conserved residues typical for β-type CAs are indicated by red font. Asterisks indicate the two amino acids that are not conserved in CAH6. (E) Schematic representation illustrating the measurement of CA activity. (TIF) [file pone.0240887.s002.tif]

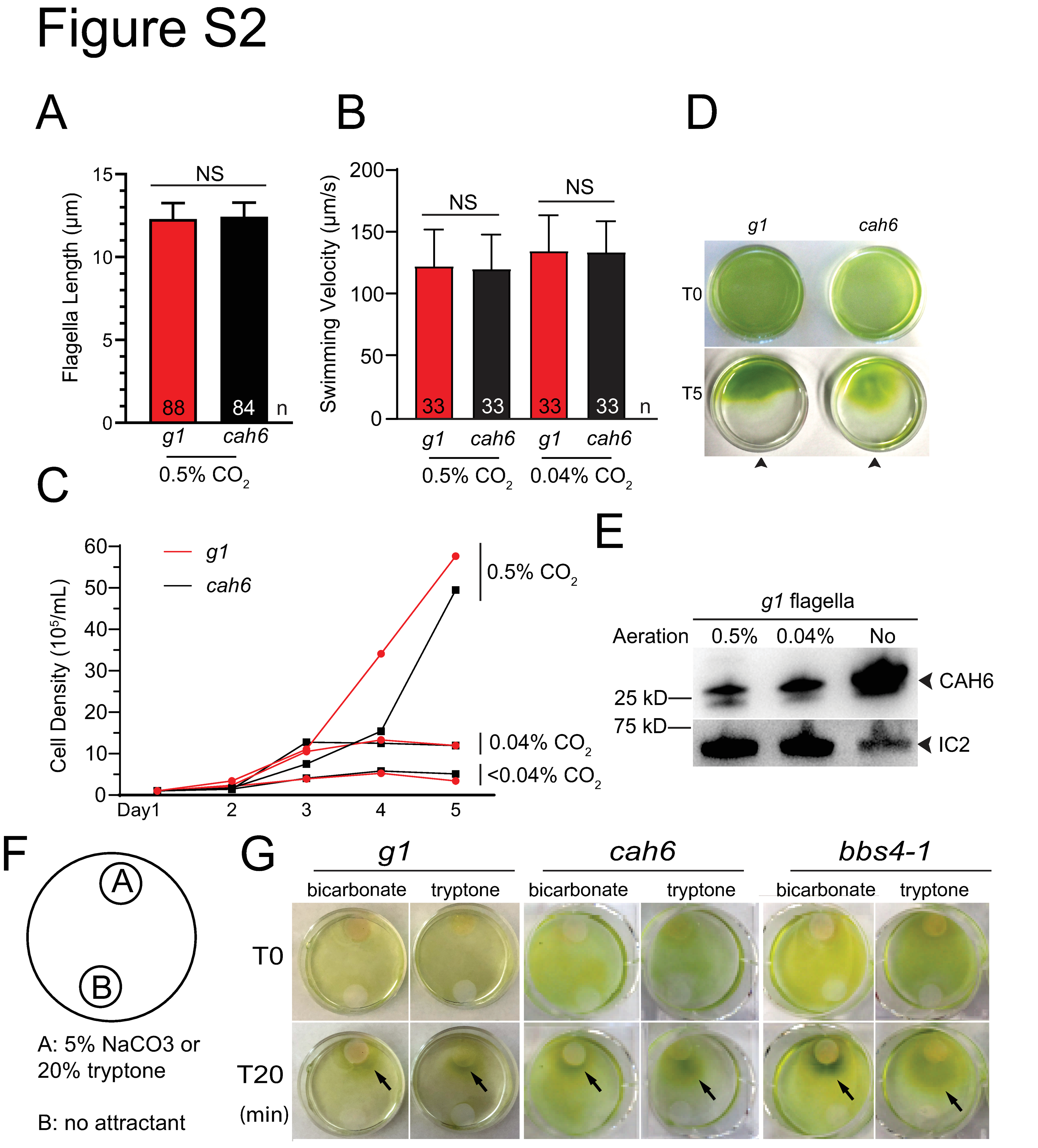

Supplement: S2 Fig — A) Flagellar length of control (g1) and the cah6 strains. The standard deviations and the number of measurements are indicated. B) Mean swimming velocity of wild-type (g1) and cah6 cells in 0.5% CO2 and 0.04% CO2 (air). C) Liquid growth curves for wild-type g1 (red circles) and cah6 (black squares) in 5% CO2, 0.04% CO2 (air) and <0.04% CO2. D) Phototaxis assay of control (g1) and cah6. The time of light exposure (5 mins) and direction of the light (arrowheads) are indicated. E) Western blot analysis of flagella for CAH6 isolated from wild-type (g1) cells aerated for 24 hours under high (0.5%), low (0.04%) and no aeration with CO2 conditions. Anti-IC2 was used as a loading control. F) Schematic presentation of the chemotaxis assay. Cells in M-medium were placed into a small Petri dish or 6-well cell culture plate and agar plaques (A, B) containing either 5% sodium bicarbonate or 20% tryptone (A) or no attractant (B) were added. G) Chemotaxis assay showing g1, cah6 and bbs4-1 before (T0) and after a 20-minute incubation in the dark (T20). Accumulated cells are indicated by the arrows. The following results were noted in repeat experiments (positive chemotaxis/no chemotaxis): g1 aerated with CO2 (0/9), g1 without aeration (5/5), cah6 (4/3), bbs4-1 (1/2), and bbs1 (2/1). (TIF) [file pone.0240887.s003.tif]

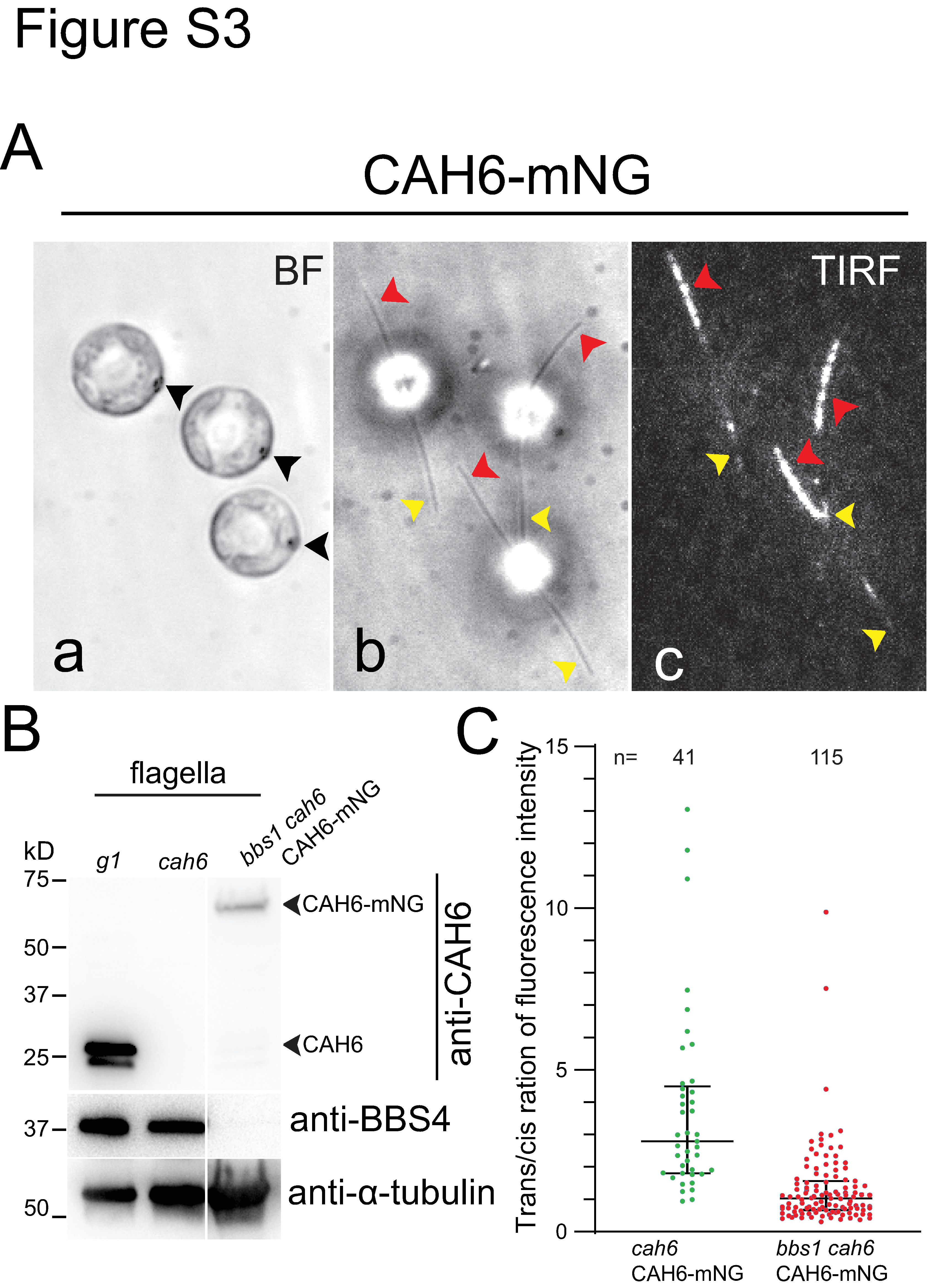

Supplement: S3 Fig — A) Brightfield (BF) and TIRFM images of live g1 CAH6-mNG cells. The two focal planes show flagella and eyespot (indicated with black arrowheads). Trans-flagella are indicated with red arrowheads and cis-flagella with yellow arrowheads. Bar = 2μm. B) Western blot analysis of isolated flagella (Flagella) from wild-type g1, cah6 and bbs1 cah6 CAH6-mNG were probed with anti-CAH6 and anti-BBS4. Antibodies to acetylated tubulin were used as a loading control. C) Scatter plot showing the ratios between the trans- and cis-flagellum of control and bbs1 cells; see Fig 3D for presentation as a histogram. (TIF) [file pone.0240887.s004.tif]

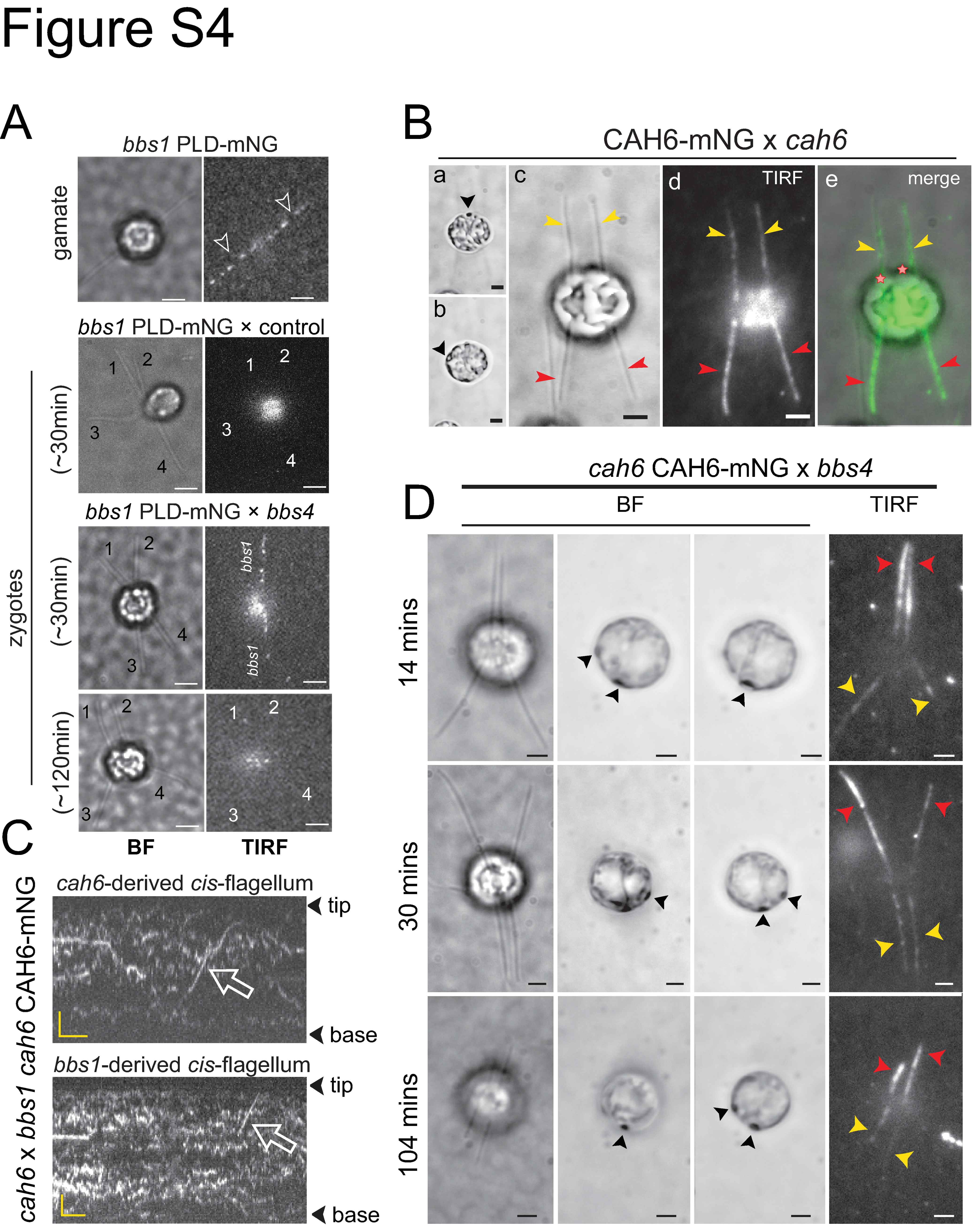

Supplement: S4 Fig — A) Brightfield (BF) and TIRFM images of a bbs1 PLD-mNG gametes and live dikaryons from a matings between bbs1 PLD-mNG and control (g1) or bbs4. In bbs1 PLD-mNG x bbs4 zygotes, both parental strains lack intact BBSomes and BBSomes need to be assembled de novo after cell fusion delaying the export of PLD-mNG. Indeed, an abnormal presence of PLD-mNG was observed in two of the four flagella in a subset of the zygotes analyzed at ≤ 30 min whereas PLD-mNG was essentially absent from all four flagella at the later time points (>30 min). Bar = 2 μm. B) Brightfield (BF) and TIRFM images of a live dikaryon from a mating between g1 CAH6-mNG and cah6 gametes. Trans-flagella are indicated with red arrowheads and cis-flagella with yellow arrowheads. Bar = 2 μm. C) Kymograms showing CAH6-mNG dynamics in the flagella of cah6 × bbs1 cah6 CAH6-mNG zygotes. Open arrows indicate transport of CAH6-mNG via anterograde IFT. D) Brightfield (BF) and TIRFM images of live dikaryons from a mating between cah6 CAH6-mNG and bbs4 gametes. The two focal planes show the flagella and the eyespot (indicated with black arrowheads). Trans-flagella are indicated with red arrowheads and cis-flagella with yellow arrowheads. Bar = 2 μm. (TIF) [file pone.0240887.s005.tif]
