# Supplementary material for: The BBSome restricts entry of tagged carbonic anhydrase 6 into the cis-flagellum of Chlamydomonas reinhardtii
Source: PLoS One. 2020 Oct 29;15(10):e0240887. doi: 10.1371/journal.pone.0240887 (PMC7595284; doi:10.1371/journal.pone.0240887)
Supplement: S1 Raw images — (DOCX) [file pone.0240887.s007.docx]

S1_raw_images Yu et al.

Fig. 1B and S1B

antibody: anti-CAH6 (first incubation); loading order: see Fig. S1B


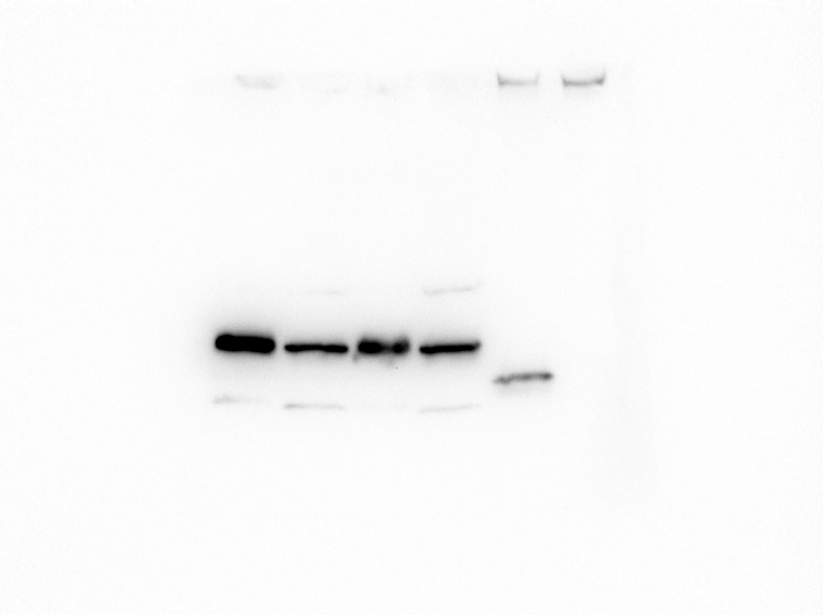


antibody: anti-IFT81 (second incubation)


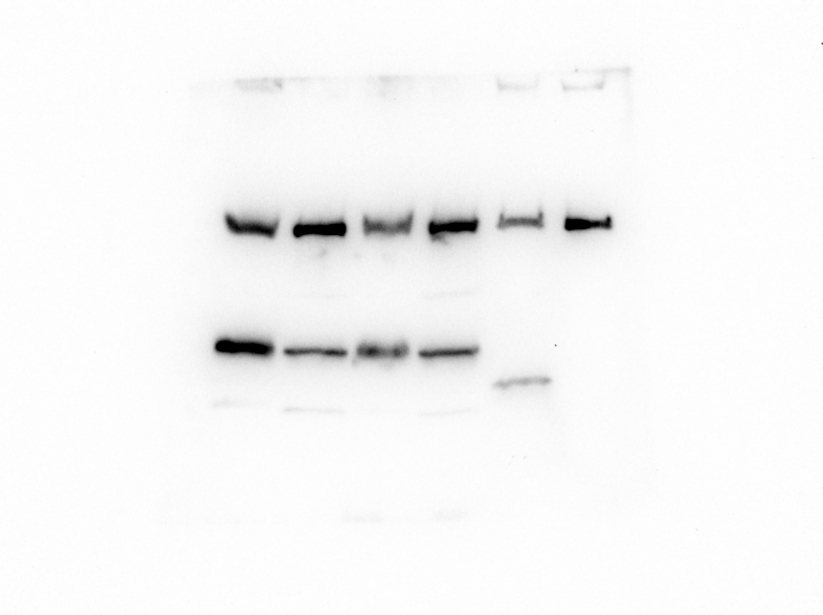


Fig2 E

Anti-CAH6 (loading order: same as in figure)


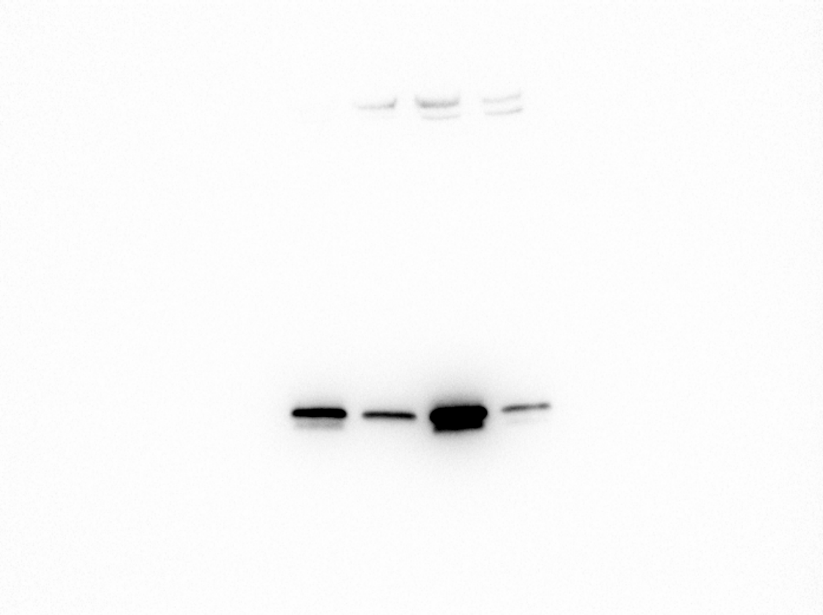


Anti-IC2


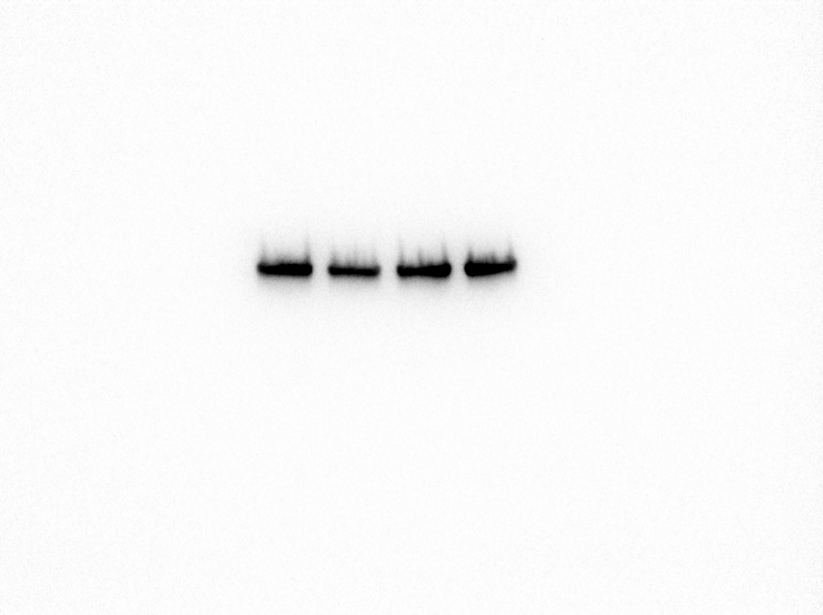


Fig. S2E

Anti-CAH6 (loading order: same as in figure)


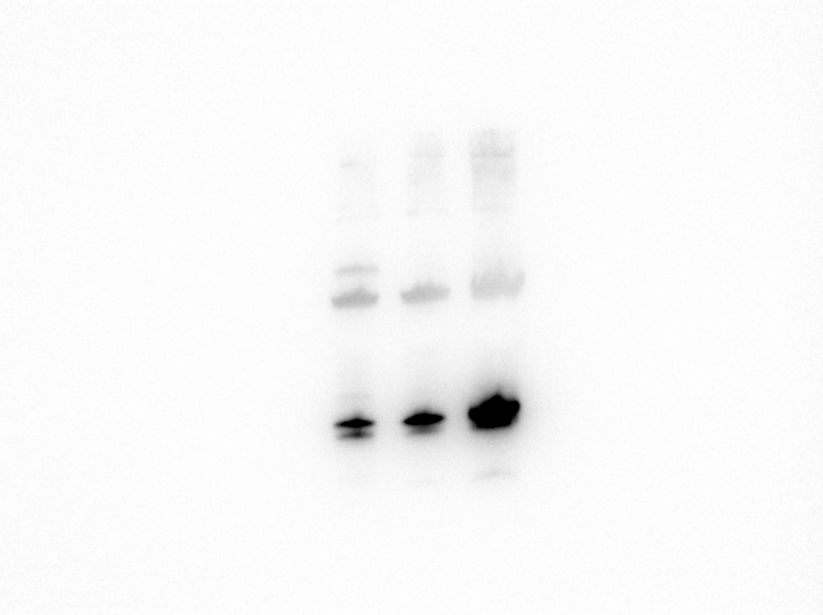


Anti-IC2


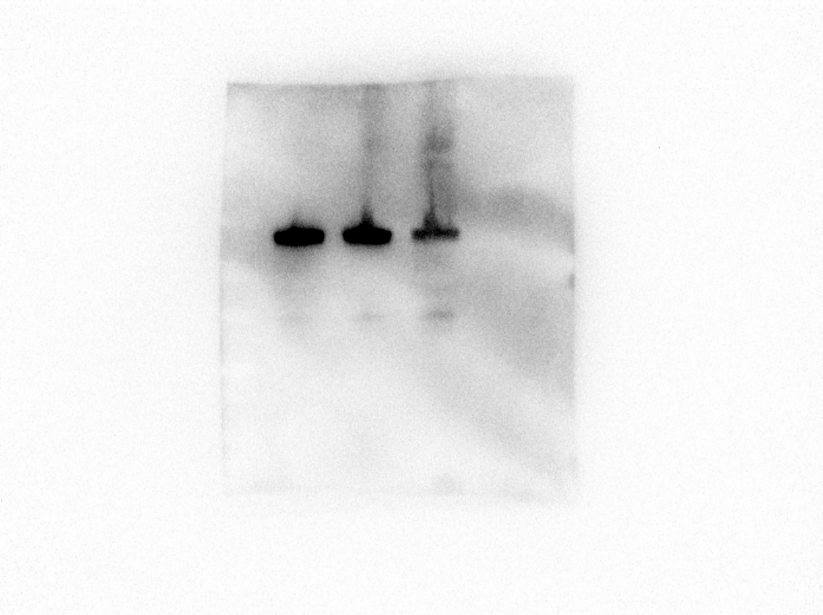


Fig. S3B

Anti-CAH6


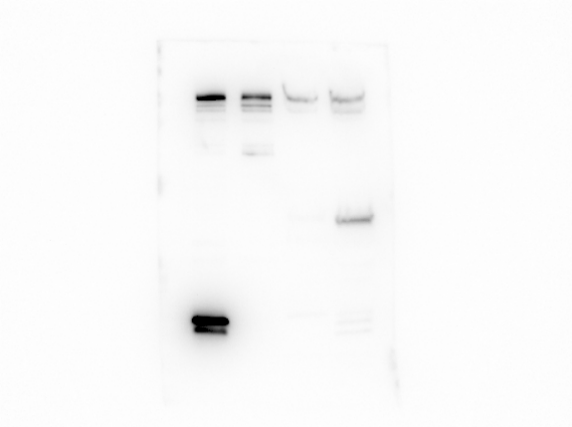


X

Anti-BBS4

X


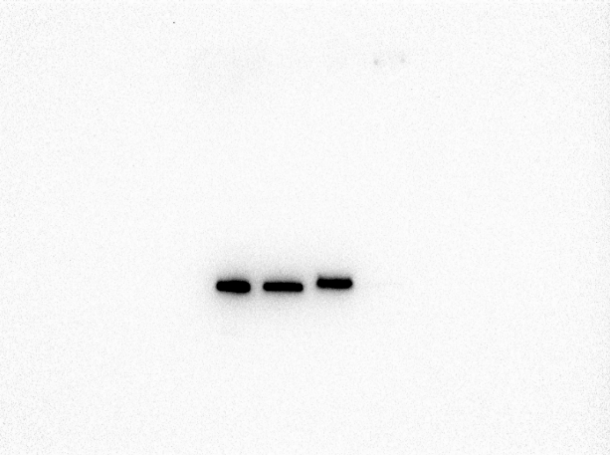


Anti-alpha-tubulin


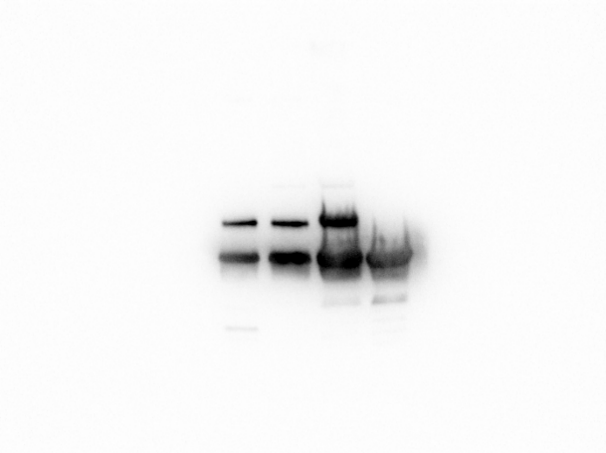


X

(Note: lane 3 showing the cah6 CAH6-mNG strain was cut out since the strain showed very low expression levels of the transgene at the time the western blot was performed)
